# Thrombospondin‐1 Regulates Trophoblast Necroptosis via NEDD4‐Mediated Ubiquitination of TAK1 in Preeclampsia

**DOI:** 10.1002/advs.202309002

**Published:** 2024-04-03

**Authors:** Haoyue Hu, Jing Ma, You Peng, Rixuan Feng, Chenling Luo, Minyi Zhang, Zixin Tao, Lu Chen, Tao Zhang, Wenqian Chen, Qian Yin, Jinguo Zhai, Jun Chen, Ailan Yin, Chi Chiu Wang, Mei Zhong

**Affiliations:** ^1^ Department of Obstetrics and Gynecology Nanfang Hospital Southern Medical University Guangzhou Guangdong 510515 China; ^2^ Guangzhou Key Laboratory of Forensic Multi‐Omics for Precision Identification School of Forensic Medicine Southern Medical University Guangzhou Guangdong 510515 China; ^3^ School of Nursing Southern Medical University Guangzhou Guangdong 510515 China; ^4^ Department of Epidemiology School of Public Health Southern Medical University Guangzhou Guangdong 510515 China; ^5^ Department of Obstetrics and Gynecology Guangzhou First People's Hospital School of Medicine South China University of Technology Guangzhou Guangdong 510180 China; ^6^ Department of Obstetrics and Gynaecology; Li Ka Shing Institute of Health Sciences; School of Biomedical Sciences; Chinese University of Hong Kong‐Sichuan University Joint Laboratory in Reproductive Medicine; The Chinese University of Hong Kong Hong Kong SAR NT China

**Keywords:** necroptosis, preeclampsia, thrombospondin‐1, trophoblast, ubiquitination

## Abstract

Preeclampsia (PE) is considered as a disease of placental origin. However, the specific mechanism of placental abnormalities remains elusive. This study identified thrombospondin‐1 (THBS1) is downregulated in preeclamptic placentae and negatively correlated with blood pressure. Functional studies show that THBS1 knockdown inhibits proliferation, migration, and invasion and increases the cycle arrest and apoptosis rate of HTR8/SVneo cells. Importantly, THBS1 silencing induces necroptosis in HTR8/SVneo cells, accompanied by the release of damage‐associated molecular patterns (DAMPs). Necroptosis inhibitors necrostatin‐1 and GSK′872 restore the trophoblast survival while pan‐caspase inhibitor Z‐VAD‐FMK has no effect. Mechanistically, the results show that THBS1 interacts with transforming growth factor B‐activated kinase 1 (TAK1), which is a central modulator of necroptosis quiescence and affects its stability. Moreover, THBS1 silencing up‐regulates the expression of neuronal precursor cell‐expressed developmentally down‐regulated 4 (NEDD4), which acts as an E3 ligase of TAK1 and catalyzes K48‐linked ubiquitination of TAK1 in HTR8/SVneo cells. Besides, THBS1 attenuates PE phenotypes and improves the placental necroptosis in vivo. Taken together, the down‐regulation of THBS1 destabilizes TAK1 by activating NEDD4‐mediated, K48‐linked TAK1 ubiquitination and promotes necroptosis and DAMPs release in trophoblast cells, thus participating in the pathogenesis of PE.

## Graphical abstract

The down‐regulation of THBS1 can destabilize TAK1 via NEDD4‐mediated, K48‐linked TAK1 ubiquitination, resulting in the activation of the necroptosis and DAMPs release in trophoblast cells, thus participating in the pathogenesis of PE.

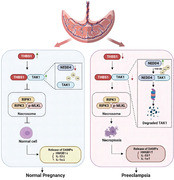

## 1 Introduction

Preeclampsia (PE) is a pregnancy‐specific syndrome characterized by hypertension, proteinuria, and other multiple organ dysfunctions at or after 20 weeks of gestation.^[^ ^1^ ^]^ It approximately complicates 2–8% of pregnancies globally and is the leading cause of maternal and perinatal morbidity and mortality.^[^ ^2^ ^]^ To date, there is no effective prevention strategy or treatment modality for PE. PE is also considered an emblematic disease of placental origin because the symptoms of PE are relieved only after the removal of the placenta, suggesting that the placenta plays a central role in PE pathogenesis.^[^ ^3^ ^]^ Although oxidative stress, trophoblast dysfunction, and impaired uterine spiral artery remodeling are thought to be the leading causes of PE, the precise etiology and pathogenesis of PE still remain elusive.^[^ ^4^ ^]^ Therefore, identifying the potential pathogenic factors of PE in the placenta can help to comprehensively understand the occurrence and development of the disease.

Thrombospondin‐1 (THBS1), which belongs to the thrombospondin family, was first identified as a glycoprotein secreted by platelets.^[^ ^5^ ^]^ THBS1 is a critical mediator of hemostasis that promotes platelet activation by modulating the inhibitory cyclic adenosine monophosphate (cAMP) signaling pathway.^[^ ^6^ ^]^ Furthermore, it is a multifunctional matricellular protein implicated in the regulation of a wide range of physiological and pathological processes, such as tissue remodeling, wound healing, angiogenesis, and inflammation, by binding to other receptors and extracellular ligands.^[^ ^7^, ^8^ ^]^ Recent studies have revealed an association between THBS1 and PE. In a case‐control study, researchers found that maternal serum THBS1 levels were significantly lower in the PE group than in the healthy pregnancy group, suggesting that THBS1 may be a new biomarker for the detection and severity of PE.^[^ ^9^ ^]^ Lower circulating THBS1 levels have also been observed in HELLP syndrome, which is a severe form of PE.^[^ ^10^ ^]^ Nevertheless, much less is known about the specific mechanisms underlying THBS1 downregulation in PE.

Necroptosis, a novel form of programmed cell death (PCD), depends on the formation and activation of the necrosome complex consisting of receptor‐interacting kinase 1 (RIPK1), RIPK3, and phosphorylated mixed lineage kinase domain‐like protein (p‐MLKL).^[^ ^11^ ^]^ The release of damage‐associated molecular patterns (DAMPs) is a typical feature of necroptosis, which triggers inflammatory signaling cascades and disrupts cellular homeostasis.^[^ ^12^ ^]^ A panoply of candidate DAMPs, such as HMGB1, IL‐1α, and IL‐33, have now been widely reported in the context of non‐infection.^[^ ^13^ ^]^ These DAMPs are produced intracellularly, thus preventing surveillance by the immune system. The DAMPs‐mediated propagation of systemic inflammation caused by necroptosis is also known as “necroinflammation”.^[^ ^14^ ^]^ Although multiple lines of evidence have proven the relationship between trophoblast necroptosis and PE, the exact mechanism still needs to be further clarified.^[^ ^15^ ^]^ In addition to the two‐stage (poor placentation and maternal endothelial dysfunction) model of PE, placenta‐specific inflammation is also a major component of the responses that lead to PE.^[^ ^16^ ^]^ Notably, the inflammatory mode in PE is more akin to sterile inflammation mediated by substances such as DAMPs rather than inflammation elicited by bacteria and viruses.^[^ ^17^, ^18^ ^]^ However, it remains poorly understood whether trophoblasts undergo necroptosis and release DAMPs to participate in occurrence and development of PE.

Transforming growth factor β‐activated kinase 1 (TAK1) is a serine/threonine kinase identified as a member of the mitogen‐activated protein kinase (MAPK) kinase kinase (MAPKKK) family. It mediates a wide range of biological processes through the regulation of the MAPK and nuclear factor‐κB (NF‐κB) signaling pathways.^[^ ^19^, ^20^ ^]^ It has been identified as a central modulator of necroptosis quiescence.^[^ ^21^ ^]^ TAK1 deficiency results in the activation of RIPK1 and the formation of the RIP1‐RIP3‐FADD necroptotic complex.^[^ ^22^ ^]^ Downregulation of TAK1 in chronic hypertensive rats induces neuronal apoptosis and necroptosis through an RIPK1‐dependent mechanism.^[^ ^23^ ^]^ Further, numerous studies have demonstrated the importance of post‐translational modifications (PTMs), especially TAK1 ubiquitination, in the regulation of TAK1.^[^ ^24^ ^]^ Ubiquitination is a dynamic process mediated by the ubiquitin‐activating enzyme (E1), ubiquitin‐conjugating enzyme (E2), and ubiquitin ligase (E3). This process can be reversed using a series of deubiquitinating enzymes (DUBs).^[^ ^25^ ^]^ A recent study suggested that the E3 ligase TRIM56 induces the ubiquitination of TAK1 by enhancing the M1‐linked polyubiquitin chains of TAK1.^[^ ^26^ ^]^ Another study reported that the E3 ligase CHIP interacts with TAK1 and targets it for K63‐linked ubiquitination.^[^ ^27^ ^]^ Although the relationship between TAK1 and necroptosis or ubiquitination has been well‐explored, its role in PE has yet to be investigated.

Our study aimed to assess the role of THBS1 in PE. We found a significant downregulation of THBS1 in preeclamptic placentae. Importantly, we found that THBS1 knockdown triggered necroptosis in trophoblasts. Mechanistically, THBS1 silencing up‐regulated the expression of neuronal precursor cell‐expressed developmentally downregulated 4 (NEDD4), thereby targeting TAK1 via K48‐linked polyubiquitination to promote its degradation. Overall, our findings suggest that THBS1 is a promising pharmaceutical target for PE treatment.

## 2 Result

### 2.1 THBS1 was Down‐Regulated in Severe Preeclamptic Placentae

**Figure 1:**
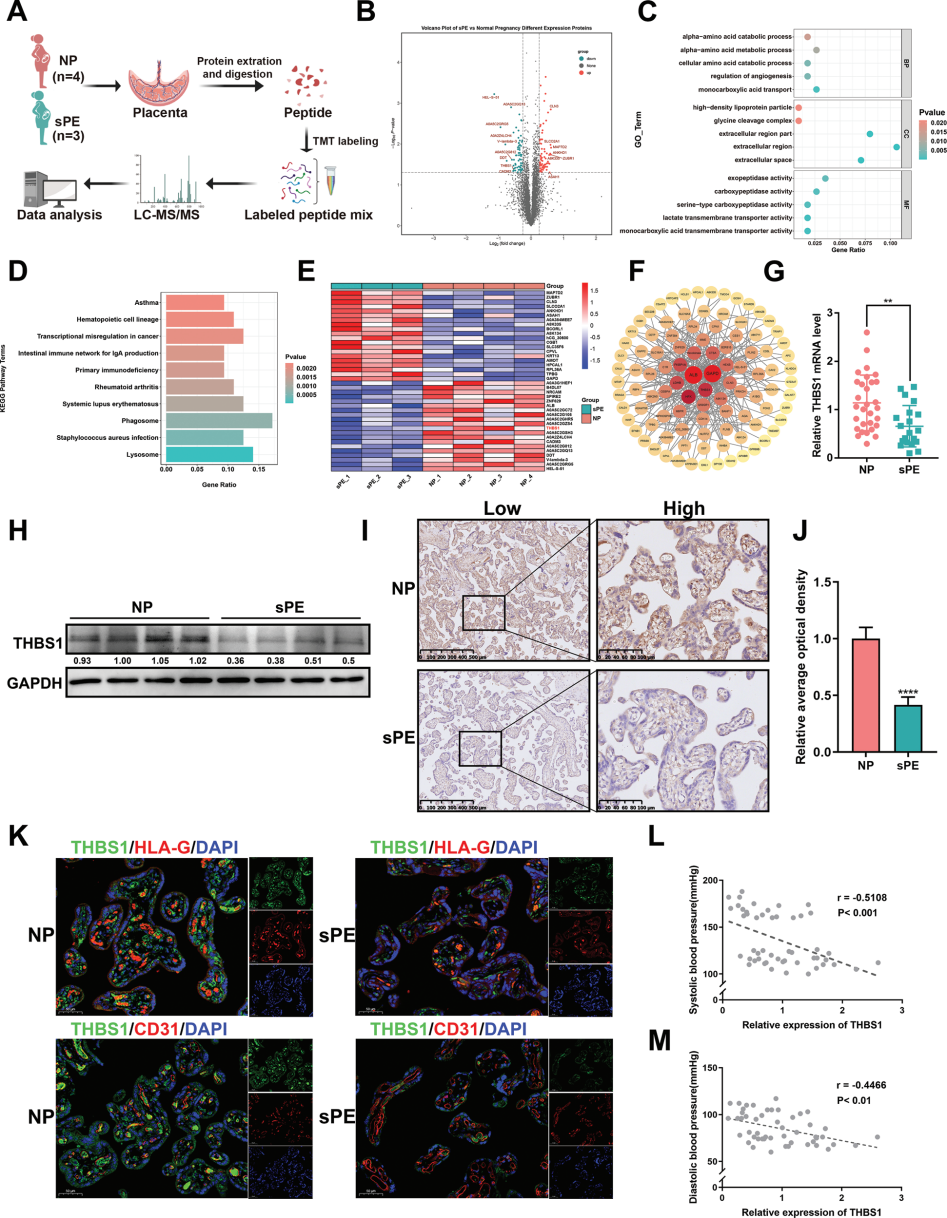
THBS1 was down‐regulated in severe preeclamptic placentae. A) Schematic representation of placental sampling and tandem mass tag (TMT)‐based quantitative proteomics profiling. B) Volcano plot showing the differentially expressed proteins (DEPs) of the placentae between normal pregnancy (NP, *n* = 4) and severe preeclampsia (sPE, *n* = 3) using TMT‐based quantitative proteomics. C) GO enrichment analysis regarding the DEPs of the placentae between the NP and sPE groups. D) KEGG enrichment analysis regarding the DEPs of the placentae between the NP and sPE groups. E) Heatmap of the top 20 DEPs that were up‐ or down‐regulated in the placentae of the sPE group. THBS1 was highlighted in red. F) The protein‐protein interaction (PPI) network analysis for the DEPs. G) RT‐qPCR analysis of THBS1 mRNA expression in NP (*n* = 30) and sPE (*n* = 20) placental tissues. H) Western blot analysis of THBS1 protein levels in the sPE and NP placentae. *n*  = 4 each group. I) The expression of THBS1 in the placental tissues of the two groups evaluated using immunohistochemistry (IHC). Scale bar = 500 µm. Representative high‐magnification images for each group are shown on the right side. Scale bar = 100 µm. J) Quantification of the IHC staining was performed using ImageJ software, *n* = 5 each group. K) Representative images of THBS1 and HLA‐G or CD31 co‐immunofluorescence staining in the sPE and NP placentae. Scale bar = 50 µm. *n*  = 3 biologically independent experiments. L,M). Spearman correlation analysis between systolic or diastolic blood pressure and the relative expression of THBS1. **, *p* < 0.01; ****, *p* < 0.0001.

To identify the key pathogenic molecules in the placentae of patients with preeclampsia, TMT‐based quantitative proteomics was performed on seven placental samples: three from patients with severe preeclampsia (sPE) and four from normal pregnant (NP) women (**Figure** **1**A). The clinical patient information is shown in **Table** **1**. There are 74 up‐regulated and 66 down‐regulated proteins (sPE/NP fold change >1.2 or <0.83, *p* <0.05) were identified as significant DEPs (Figure 1B). GO enrichment analysis demonstrated that the DEPs between the two groups were involved in several biological processes, cellular components, and molecular functions (Figure 1C). KEGG enrichment analysis showed that pathways, such as phagosome and lysosome pathways, were altered significantly between the two groups (Figure 1D). A heat map was used to illustrate the top 20 proteins that were up‐ or down‐regulated in the placentae of the sPE group (Figure 1E). The PPI network revealed the links of the DEPs and found that proteins, such as GAPD, ALB, THBS1, and LDHB, were in the core location (Figure 1F). Among these, THBS1 was one of the DEPs that was significantly down‐regulated in the placentae of patients with sPE, and it has the largest number of GO entries in the GO database (Table S1, Supporting Information).Therefore, THBS1 was selected as the primary research object in this study.

**Table 1:** Characteristics of placental samples for TMT‐based quantitative proteomic.

Sample	Age [years]	BMI [kg m^−2^]	Gestational age [days]	Systolic blood pressure [mmHg]	Diastolic blood pressure [mmHg]	Proteinuria	Infant birth weight [g]
NP_1	28	22.641	276	100	68	–	2720
NP_2	35	24.974	271	126	67	–	4090
NP_3	34	20.343	266	123	70	–	3840
NP_4	28	18.750	269	110	76	–	2660
sPE_1	30	22.266	266	168	105	++	2550
sPE_2	32	22.074	263–	182	112	++++	2220
sPE_3	33	19.133	272	173	96	++++	3340

To further confirm the differential expression of THBS1 in the placentae of the sPE and NP groups, we performed RT‐qPCR and western blot assays. Characteristics of the 50 placental samples were summarized in **Table** **2**. The results showed that compared with the NP group, the mRNA (Figure 1G) and protein levels (Figure 1H) of THBS1 were significantly lower in the placentae of patients with sPE. The expression and localization of THBS1 were further validated using IHC (Figure 1I,J) and double‐staining immunofluorescence analyses (Figure 1K). The results showed that THBS1 was more highly expressed in preeclamptic placentae than in normal placentae, which was consistent with the results of RT‐qPCR and western blot. Furthermore, HLA‐G and CD31 were used as markers for extravillous trophoblasts (EVTs) and vascular endothelial cells, respectively. Our data revealed that THBS1 was prominently localized in the EVT but not in the vascular endothelial cells of the placentae, suggesting that THBS1 might participate in the occurrence and development of PE through EVT dysfunction. Moreover, THBS1 in the placentae was negatively correlated with the clinical characteristics of PE, such as increased systolic and diastolic blood pressure (Figure 1L,M).

**Table 2:** Characteristics of the 50 placental samples.

Characteristics	Normal Pregnancy [*n* = 30]	Serve preeclampsia [*n* = 20]	*p* value
Maternal age [years]	29.667±3.818	31.600±3.648	0.081
BMI [kg m^−2^]	26.070±3.942	27.570±3.896	0.192
Gestational age [days]	272.367±7.318	265.300±10.352	0.007
Systolic blood pressure [mmHg]	114.600±7.040	168.850±8.222****	<0.001
Diastolic blood pressure [mmHg]	73.767±7.205	102.700±7.124****	<0.001
Proteinuria	–	+∼++++	–
Infant birth weight [g]	3156.333±413.234	2798.000±544.790****	<0.001

Data are shown as mean± SD.

### 2.2 THBS1 Deficiency Induced Dysfunctions in Trophoblast Cells

**Figure 2:**
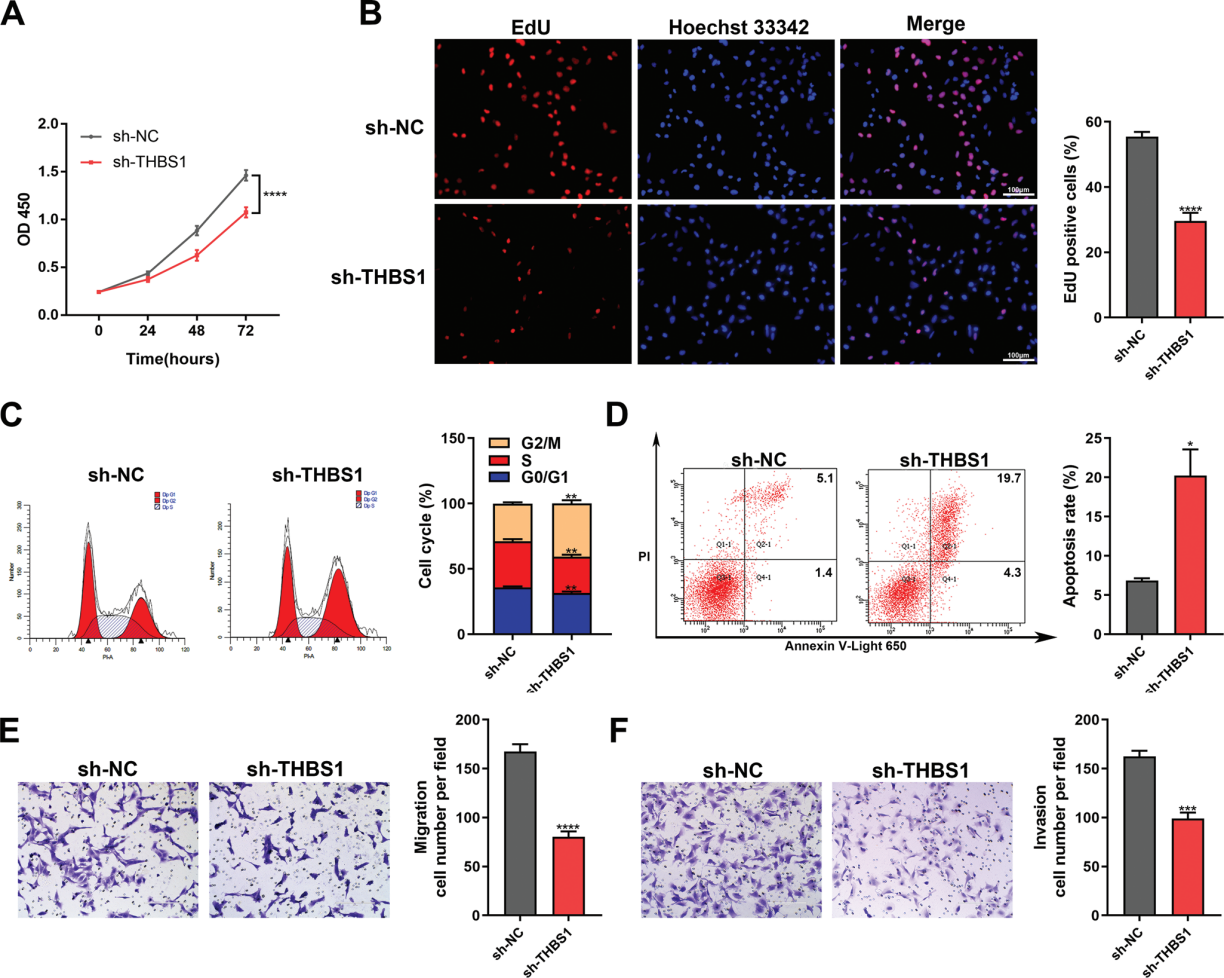
THBS1 deficiency induced dysfunctions in trophoblast cells. A) CCK8 assay was used to detect the cell viability of sh‐NC and sh‐THBS1 stable‐transfected HTR8/SVneo cells at specific time points. *n*  = 5 biologically independent experiments. B) EdU assay was used to determine the proliferation of sh‐NC and sh‐THBS1 stable‐transfected HTR8/SVneo cells. Left panel: representative images of the EdU assay in each group; right panel: statistics of EdU‐positive cells. *n*  = 3 biologically independent experiments. C) The cell cycles of sh‐NC and sh‐THBS1 stable‐transfected HTR8/SVneo cells assessed using flow cytometry. Left panel: representative images of flow cytometry in each phase; right panel: quantification of cells in each phase. *n*  = 3 biologically independent experiments. D) Apoptosis of sh‐NC and sh‐THBS1 stable‐transfected HTR8/SVneo cells assessed using the Annexin V‐Light 650/PI Apoptosis Detection Kit and flow cytometry. Left panel: representative histograms of apoptotic cells in each group; right panel: quantification of apoptotic cells in each group. *n*  = 3 biologically independent experiments. E) The migratory capability of sh‐NC and sh‐THBS1 stable‐transfected HTR8/SVneo cells examined using a transwell assay. Left panel: representative images of migratory cells in each group; right panel: quantification of cells in each group. *n*  = 3 biologically independent experiments. F) The invasive capability of sh‐NC and sh‐THBS1 stable‐transfected HTR8/SVneo cells examined using a transwell assay. Left panel: representative images of cells in each group; right panel: quantification of invasive cells in each group. *n*  = 3 biologically independent experiments. *, *p* < 0.05; **, *p* < 0.01; ***, *p* < 0.001, ****, *p* < 0.0001.

HTR8/SVneo cells are a classical in vitro model of EVT, which were used to ascertain the effect of THBS1 on trophoblasts in this study. First, we constructed stable knockdown (sh‐) or overexpression (OE‐) of THBS1 using lentiviruses in HTR8/SVneo cells (Figure S1, Supporting Information). The results of the CCK8 assay (**Figure** **2**A) and EdU staining (Figure 2B) showed that THBS1 knockdown inhibited the viability and proliferation of HTR8/SVneo cells. Flow cytometry assays showed that silencing THBS1 induced cell cycle arrest at the G2/M phase and reduced the percentage of cells in the S and G0/G1 phases (Figure 2C). It also notably increased the rate of cellular apoptosis (Figure 2D). Moreover, a transwell assay was used to evaluate the effects of THBS1 on the migration and invasion of HTR8/SVneo cells. The results showed that THBS1 knockdown inhibited both the migratory and invasive abilities of the cells (Figure 2E,F).

In addition, THBS1 overexpression promoted the proliferation of HTR8/SVneo cells (Figure S2A,B, Supporting Information). However, it did not significantly alter the cell cycle (Figure S2C, Supporting Information), apoptosis (Figure S2D, Supporting Information), or the migratory and invasive abilities of the cells (Figure S2E,F, Supporting Information).

### 2.3 Inhibition of THBS1 Induced Necroptosis but not Caspase‐Dependent Apoptosis in Trophoblast Cells

**Figure 3:**
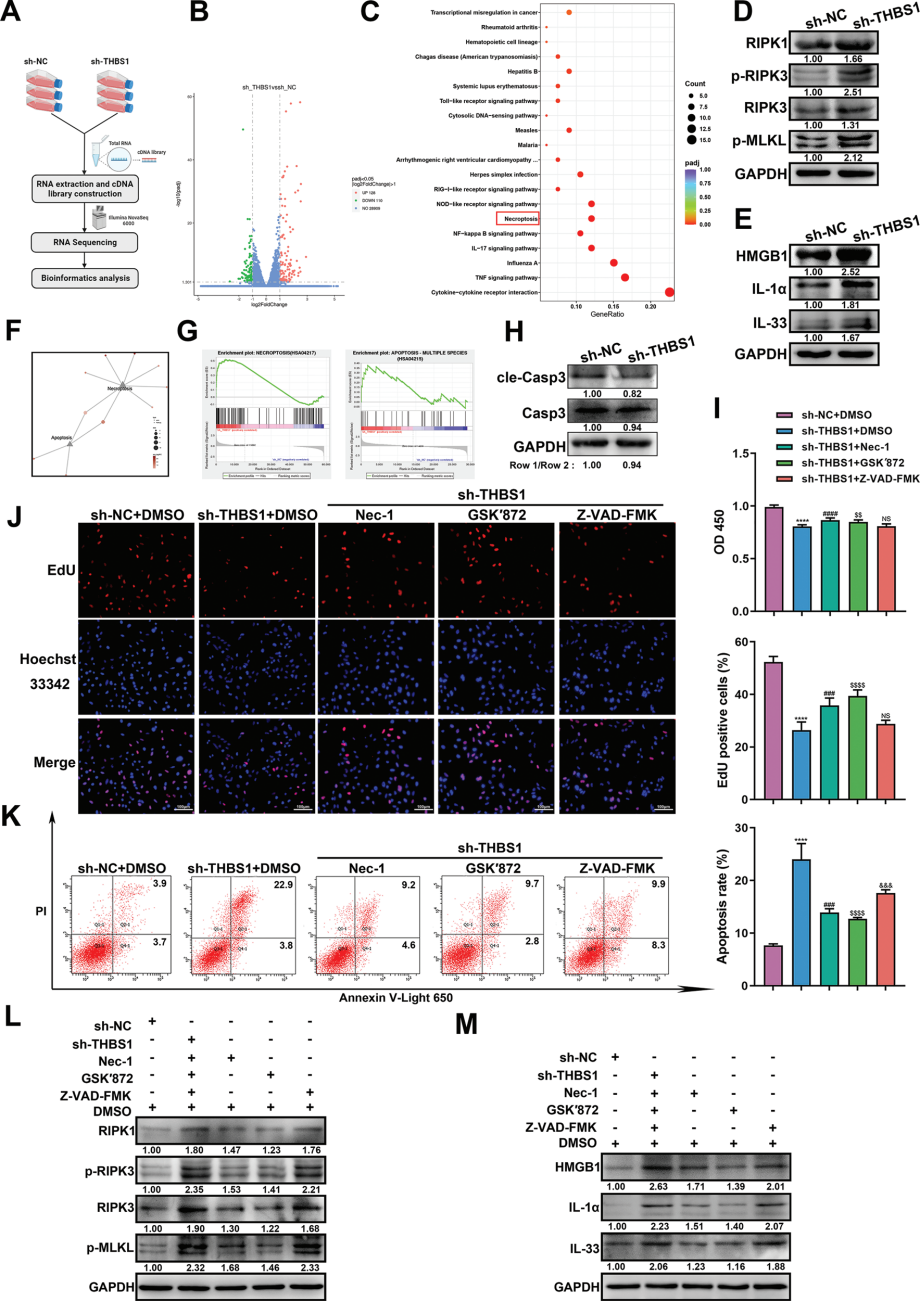
Inhibition of THBS1 induced necroptosis but not caspase‐dependent apoptosis in trophoblast cells. A) Workflow of RNA extraction and RNA‐seq of sh‐NC and sh‐THBS1 stable‐transfected HTR8/SVneo cells. B) Volcano Plot of the up‐regulated and down‐regulated genes between the sh‐NC and sh‐THBS1 groups. C) Scatter plots regarding the up‐regulated pathways following KEGG pathway analysis. D,E) Western blot analysis of necroptosis and DAMPs protein levels in the sh‐NC and sh‐THBS1 groups. *n* = 3 biologically independent experiments. F) KEGG pathway relationship network of DEGs in the necroptosis and apoptosis pathways. G) GSEA showed that the necroptosis pathway was more positively regulated than the apoptosis pathway in the sh‐THBS1 group. H) Western blot analysis of cleaved‐caspase 3 and caspase 3 protein levels. *n* = 3 biologically independent experiments. I) Cell viability was assessed using the CCK8 assay after the administration of different inhibitors. *n* = 5 biologically independent experiments. J) Cell proliferation was determined by the EdU assay after the administration of different inhibitors. Left panel: representative images of the EdU assay in each group; right panel: statistics of EdU‐positive cells. *n* = 3 biologically independent experiments. K) Cell apoptosis was assessed using the Annexin V‐Light 650/PI Apoptosis Detection Kit and flow cytometry after the administration of different inhibitors. Left panel: representative histograms of apoptotic cells in each group; right panel: quantification of apoptotic cells in each group. *n* = 3 biologically independent experiments. L,M). Western blot analysis of necroptosis and DAMPs‐related protein levels in cells treated with different inhibitors. *, sh‐THBS1 + DMSO versus sh‐NC + DMSO group. #, sh‐THBS1 + nec‐1 versus sh‐THBS1 + DMSO group. $, sh‐THBS1 + GSK′872 versus sh‐THBS1 + DMSO group. &, sh‐THBS1 + Z‐VAD‐FMK versus sh‐THBS1 + DMSO group. *, #, $, &, *p* < 0.05; **, ##, $$, &&, *p* < 0.01; ***, ###, $$$, &&&, *p* < 0.001, ****, ####, $$$$, &&&&, *p* < 0.0001. NS, no significance.

To elucidate the underlying regulatory mechanisms of THBS1 in trophoblast dysfunction, RNA‐seq was performed to identify the differential genes and pathways in the sh‐THBS1 and sh‐NC groups (**Figure** **3**A). In total, 238 DEGs were identified, including 128 up‐regulated and 110 down‐regulated genes (Figure 3B). KEGG analysis revealed that the up‐regulated genes were mainly enriched in several pathways, including the TNF signaling pathway, NF‐κB signaling pathway, Nod‐like receptor signaling pathway and necroptosis pathway (Figure 3C). Among these pathways, necroptosis attracted our attention. We further detected the expression of key molecules in the necroptosis pathway, such as RIPK1, p‐RIPK3, RIPK3, and p‐MLKL, using western blot. The results showed that the protein levels of these molecules in the sh‐THBS1 group were significantly elevated compared to those in the sh‐NC group (Figure 3D). Since necroptosis usually induces the release of DAMPs and augmentation of inflammation, we also examined the protein levels of DAMPs such as HMGB1, IL‐1α, and IL‐33. The results suggested that the expression of DAMPs was also enhanced by THBS1 knockdown (Figure 3E).

We also confirmed the expression of necroptosis and DAMPs‐related proteins in clinical samples. They were all notably up‐regulated in preeclamptic placentae (Figure S3, Supporting Information).

To confirm that necroptosis was the main cause of trophoblast dysfunction induced by THBS1 knockdown, we used KEGG pathway network analysis to compare the effects of THBS1 knockdown on necroptosis and apoptosis. We found that the number of DEGs focused on necroptosis was higher than those focused on apoptosis (Figure 3F). GSEA analysis also showed that THBS1 knockdown resulted in a remarkable up‐regulation of necroptosis but had little effect on apoptosis (Figure 3G). Also, THBS1 knockdown had no effect on the expression of cleaved‐caspase 3, a canonical marker of the apoptotic pathway (Figure 3H). Then, we administered necrostatin‐1 (Nec‐1; 25 µM), GSK′872 (10 µM), and Z‐VAD‐FMK (25 µM), which are specific inhibitors of RIPK1, RIPK3, and pan‐caspase, respectively, to THBS1‐knockdown HTR8/SVneo cells for 24 h. We found that both Nec‐1 and GSK′872 obviously restored the viability (Figure 3I) and proliferation (Figure 3J) of THBS1‐knockdown HTR8/SVneo cells, whereas Z‐VAD‐FMK did not. Consistently, Nec‐1 and GSK′872 reduced the apoptosis rate of THBS1‐knockdown cells, while Z‐VAD‐FMK did not improve the apoptosis rate (Figure 3K). Subsequently, we detected the expression changes of RIPK1, p‐RIPK3, RIPK3, p‐MLKL, and DAMP molecules at the protein level after the administration of Nec‐1, GSK′872, and Z‐VAD‐FMK in THBS1‐knockdown HTR8/SVneo cells. The results also suggested that Nec‐1 and GSK′872 significantly decreased the expressions of RIPK1, p‐RIPK3, RIPK3, p‐MLKL, and DAMP molecules compared with the sh‐THBS1+DMSO group. However, Z‐VAD‐FMK did not alter the expression of these proteins in THBS1‐knockdown cells (Figure 3L,M).

Since the Nod‐like receptor signaling pathway was enriched in the RNA‐seq results, we also examined the effect of THBS1 knockdown on pyroptosis. The results showed that THBS1 knockdown induced pyroptosis in HTR8/SVneo cells (Figure S4, Supporting Information).

### 2.4 THBS1 Interacted with TAK1 in Trophoblast Cells

There is growing evidence showed that Z‐nucleic acid‐binding protein 1 (ZBP1) and TAK1 act as master regulators of PANoptosis, which includes pyroptosis, apoptosis, and necroptosis.^[^ ^28^ ^]^ And the activation of ZBP1and inhibition of TAK1 can trigger necroptosis.^[^ ^22^, ^29^ ^]^ Given that we found that THBS1 knockdown caused necroptosis and pyroptosis in trophoblasts, we examined the expression of ZBP1 and TAK1 in the human placenta using The Human Protein Atlas database (www.proteinatlas.org/). We found that ZBP1 was almost not expressed in the human placenta (Figure S5A, Supporting Information), whereas TAK1 was highly expressed (Figure S5B, Supporting Information). In order to determine whether TAK1 inhibition could induce necroptosis in HTR8/SVneo cells, we knocked down the expression of TAK1 and examined its effect on necroptosis and DAMPs molecules (Figure S6, Supporting Information). The results showed that silencing of TAK1 significantly activated the levels of necroptosis and DAMPS‐related proteins.

**Figure 4:**
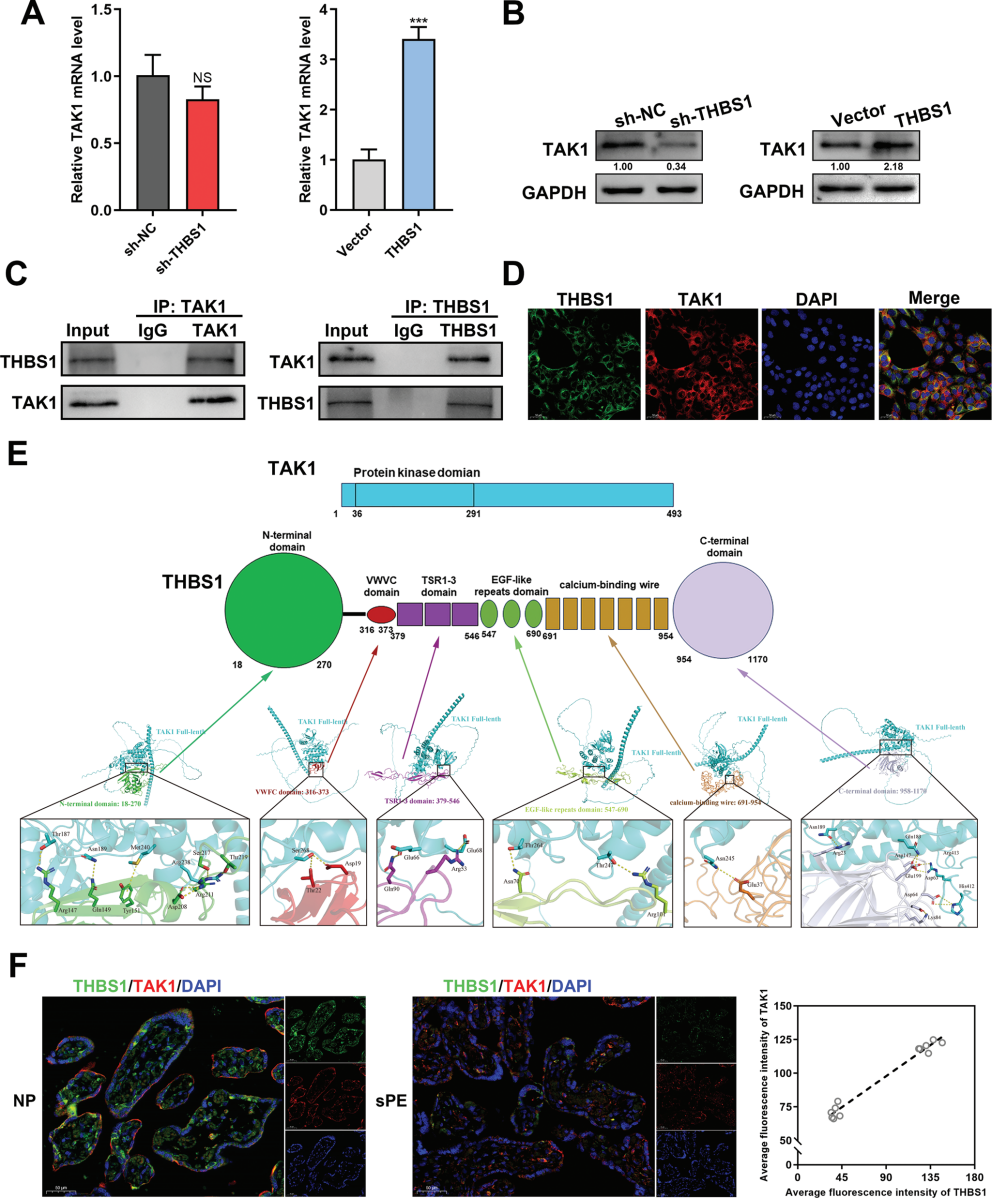
THBS1 interacted with TAK1 in trophoblast cells. A) RT‐qPCR analysis of TAK1 mRNA expression in silencing or overexpressing THBS1 in HTR8/SVneo cells. *n* = 3 biologically independent experiments. B) Western blot analysis of TAK1 protein levels in silencing or overexpressing THBS1 in HTR8/SVneo cells. *n* = 3 biologically independent experiments. C) Co‐immunoprecipitation (Co‐IP) assays were performed to analyze the interactions between THBS1 and TAK1. D) Co‐localization of THBS1 and TAK1 detected using immunofluorescence. E) Predicted binding complex models of THBS1 and TAK1 using AlphaFold2 structure prediction. Upper panel: schematic diagram of the domains of THBS1 and full‐length TAK1 proteins; lower panel: the putative binding mode between different THBS1 domains and full‐length of TAK1. F) Left panel: Representative images of THBS1 and TAK1 co‐immunofluorescence staining in the sPE and NP placentae. Scale bar = 50 µm. Right panel: Spearman's correlation analysis between the average fluorescence intensities of TAK1 and THBS1. *n* = 5 biologically independent experiments. ***, *p* < 0.001, NS, no significance.

Then, we conducted RT‐qPCR and western blot to assess the effects of THBS1 on TAK1 expression. We found that THBS1 knockdown had no significant effect on the mRNA level of TAK1 but dramatically reduced its protein level. Overexpression of THBS1 up‐regulated the expression of TAK1 at both the mRNA and protein levels (**Figure** **4**A,B). It is speculated that THBS1 regulates necroptosis by targeting TAK1. Thus, we investigated whether THBS1 could interact with TAK1. Co‐IP assays confirmed the interaction between endogenously expressed THBS1 and TAK1 in HTR8/SVneo cells (Figure 4C). Immunofluorescence staining revealed that THBS1 and TAK1 were mainly co‐localized in the cytoplasm of HTR8/SVneo cells (Figure 4D). The THBS1 protein contains six domains: the N‐terminal domain (18–270 aa), VWFC domain (316–373 aa), TSR1‐3 domain (379‐546 aa), EGF‐like repeat domain (547‐690 aa), calcium‐binding wire (691‐954 aa), and C‐terminal domain (958–1170 aa). To further elucidate the specific binding sites of the interaction between THBS1 and TAK1, we performed AlphaFold2 structure prediction of the six domains of THBS1 with full‐length TAK1. The results showed that the N‐terminal domain of THBS1 had the highest number of binding sites for TAK1 (Figure 4E).

Consistent with the in vitro data, the protein level of TAK1 was also down‐regulated in placentae of sPE (Figure S7, Supporting Information). Then we further detected the correlation between THBS1 and TAK1 in the human placentae. Double‐stained immunofluorescence analysis visually demonstrated the co‐localization of THBS1 and TAK1 in human placenta, and revealed the positive correlation between them (Figure 4F).

### 2.5 THBS1 Maintained the Stability of TAK1 in Trophoblast Cells

**Figure 5:**
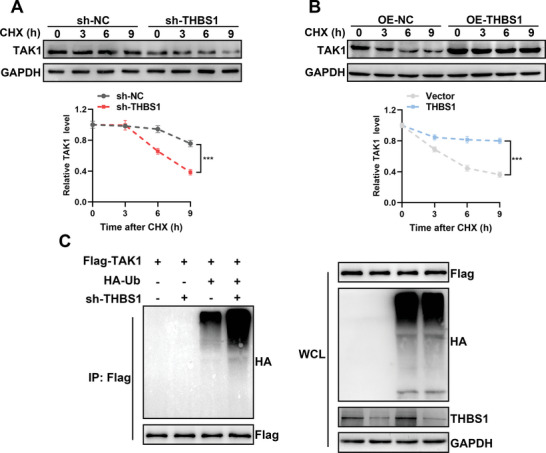
THBS1 maintained the stability of TAK1 in trophoblast cells. A,B) Degradation of TAK1 protein was measured after CHX treatment at the indicated time points in HTR8/SVneo cells with silenced or overexpressed THBS1. The stability of TAK1 was detected by western blot (upper panel) and analyzed by ImageJ software (lower panel). *n* = 3 biologically independent experiments. C) HTR8/SVneo cells stably transfected with sh‐THBS1 or sh‐NC were co‐transfected with Flag‐TAK1 and HA‐Ub. Cell lysates were immunoprecipitated with an anti‐Flag antibody, and protein production was analyzed using western blot with anti‐HA and anti‐Flag antibodies. *n* = 3 biologically independent experiments.

Next, whether THBS1 could affect the stability of TAK1 was determined in HTR8/SVneo cells. CHX was used to inhibit protein synthesis and observe the stability of TAK1. As shown, THBS1 silencing led to a rapid degradation of TAK1 (**Figure** **5**A), whereas THBS1 overexpression maintained the stability of TAK1 (Figure 5B). We further explored the role of THBS1 in TAK1 ubiquitination. The results showed that THBS1 knockdown increased the TAK1‐ubiquitin (Ub) interaction (Figure 5C). These results indicated that THBS1 silencing attenuated the stability of TAK1 by activating its ubiquitination.

### 2.6 THBS1 Knockdown Destabilized TAK1 by Activating NEDD4‐Mediated, K48‐Linked TAK1 Ubiquitination

**Figure 6:**
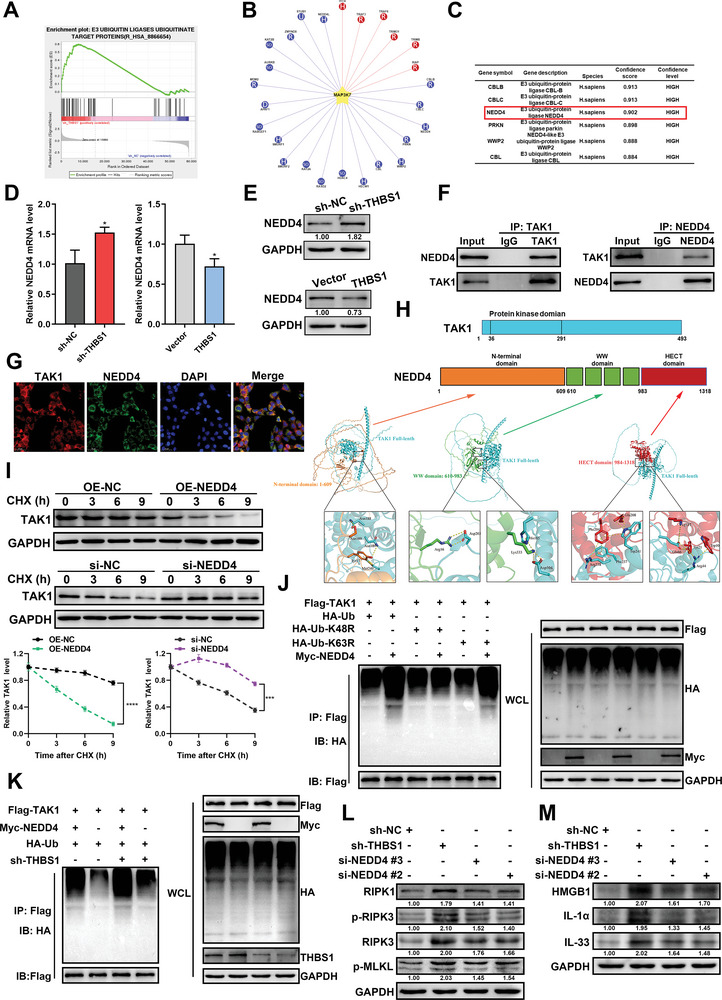
THBS1 knockdown destabilized TAK1 by activating NEDD4‐mediated, K48‐linked TAK1 ubiquitination. A) GSEA showed that E3 ubiquitin ligases ubiquitinate target proteins pathway was positively regulated in HTR8/SVneo cells that silencing THBS1. B) The existing and potential E3 ligases of TAK1 were predicted by the UbiBrowser 2.0 database. The red color represents the known E3 ligase. The blue color represents the predicted E3 ligase. C) The table of the top 6 predicted E3 ligases of TAK1. D) RT‐qPCR analysis of NEDD4 mRNA expression in silencing or overexpressing THBS1 in HTR8/SVneo cells. *n* = 3 biologically independent experiments. E) Western blot analysis of NEDD4 protein levels in silencing or overexpressing THBS1 in HTR8/SVneo cells. *n* = 3 biologically independent experiments. F) Co‐IP assays were performed to analyze the interactions between TAK1 and NEDD4. G) Co‐localization of TAK1 and NEDD4 was detected using immunofluorescence. H) Predicted binding complex models of TAK1 and NEDD4 via AlphaFold2 structure prediction. Upper panel: schematic diagram of the domains of NEDD4 and full‐length of TAK1 proteins; lower panel: the putative binding mode between different domains of NEDD4 and full‐length of TAK1. I) Degradation of the TAK1 protein was measured after CHX treatment at the indicated time points in HTR8/SVneo cells with silenced or overexpressed NEDD4. The stability of TAK1 was detected by western blot (upper panel) and analyzed by ImageJ software (lower panel). *n* = 3 biologically independent experiments. J) After HTR8/SVneo cells were co‐transfected with Flag‐TAK1, Myc‐NEDD4, and HA‐Ub or its mutants’ plasmids, the ubiquitin levels of TAK1 were detected by Co‐IP. K) HTR8/SVneo cells stably transfected with sh‐THBS1 or sh‐NC were co‐transfected with Flag‐TAK1 and Myc‐NEDD4 or its vector. The ubiquitination level of TAK1 was detected using Co‐IP. L,M) After si‐NEDD4 transfection of cells with stable THBS1 silencing, western blot analysis of necroptosis and DAMPs protein levels was performed. *n* = 3 biologically independent experiments. *, *p* < 0.05.

THBS1 primarily serves as a matricellular protein and is unlikely to directly induce TAK1 ubiquitination. Using GSEA analysis based on the Reactome database, we identified that the pathway of E3 ubiquitin ligases ubiquitinate target proteins was positively regulated in the sh‐THBS1 group in HTR8/SVneo cells (**Figure** **6**A). Based on the RNA‐seq results, we selected the top 10 E3 ubiquitin ligases that were up‐regulated in the sh‐THBS1 group (Figure S8, Supporting Information). The UbiBrowser 2.0 database was used to predict the E3 ligase of TAK (gene symbol: MAP3K7). Both 6 known and the top 20 predicted E3 ligases are shown in Figure 6B. The top 6 predicted E3 ligases ranked in Figure 6C were CBLB, CBLC, NEDD4, PRKN, WWP2, and CBL. Among these, NEDD4 was highly expressed in RNA‐seq of the sh‐THBS1 group.

The effect of THBS1 on NEDD4 expression was validated by RT‐qPCR and western blot. We found that the mRNA and protein levels of NEDD4 were up‐regulated or down‐regulated in cells with silenced THBS1 or cells overexpressing THBS1, respectively (Figure 6D,E). Thus, we speculated that THBS1 knockdown might affect the ubiquitination of TAK1 by activating the expression of NEDD4. We further investigated the interaction between NEDD4 and TAK1. Co‐IP assays demonstrated that endogenous NEDD4 and TAK1 were bound to each other in HTR8/SVneo cells (Figure 6F). In addition, NEDD4 and TAK1 were co‐localized in the cytoplasm of HTR8/SVneo cells (Figure 6G). The NEDD4 protein contains three domains: an N‐terminal domain (1–608 aa), a WW domain (608–982 aa), and an HECT domain (983–1319 aa). The results of AlphaFold2 structure prediction predicted the specific binding sites of each domain of NEDD4 to the full length of TAK1 and found that the HECT domain of NEDD4 had the most binding sites with TAK1 (Figure 6H). CHX treatment led to rapid degradation of TAK1 in HTR8/Svneo cells transfected with the NEDD4‐overexpressed plasmid. However, along with a reduction in NEDD4 levels, TAK1 was degraded slowly (Figure 6I).

To investigate the type of NEDD4‐mediated ubiquitination of TAK1, we used vectors expressing HA‐tagged wild‐type ubiquitin (HA‐Ub) and its mutants (HA‐Ub‐K48R and HA‐Ub‐K63R, in which lysine was substituted with arginine at positions 48 and 63, respectively). K63‐linked ubiquitination preferentially degrades substrates through the autophagy/lysosome pathway, whereas K48‐linked ubiquitination generally degrades substrates through the proteasomal pathway.^[^ ^30^ ^]^ We found that NEDD4 enhanced the binding of TAK1 to Ub, but reduced the binding of Ub to TAK1 when the HA‐Ub‐K48R plasmid was simultaneously transfected (Figure 6J). In summary, these data confirmed that NEDD4 directly interacted with TAK1 and catalyzed K48‐linked ubiquitination of TAK1.

Furthermore, the degradation of TAK1 by down‐regulated‐THBS1 was reduced in the absence of NEDD4, suggesting that THBS1 knockdown destabilized TAK1 by activating NEDD4‐mediated TAK1 ubiquitination (Figure 6K). Additionally, we constructed an siRNA of NEDD4 and determined its knockdown efficiency (Figure S9, Supporting Information). Two NEDD4 siRNAs were selected for subsequent experiments. We examined whether NEDD4 knockdown could reverse the silencing of THBS1‐mediated necroptosis and the expression of DAMPs‐related proteins in HTR8/SVneo cells. The results showed that NEDD4 knockdown reduced the levels of these proteins activated by THBS1 silencing (Figure 6L,M).

### 2.7 Supplementation with THBS1 Ameliorated PE Phenotypes and Placental Necroptosis in Pregnant Mice

**Figure 7:**
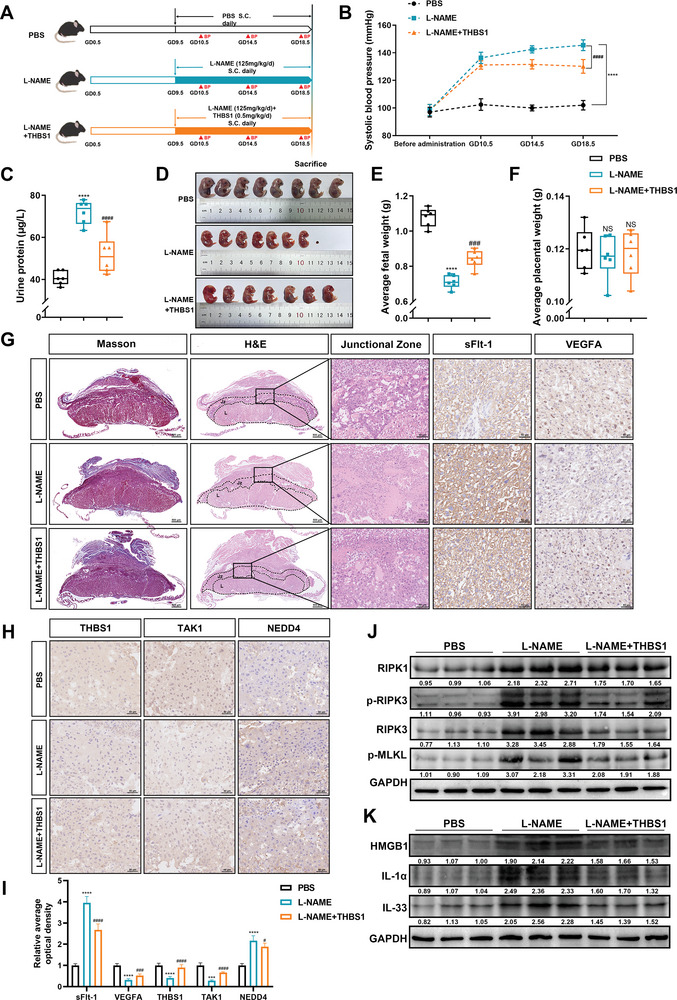
Supplementation with THBS1 ameliorated PE phenotypes and placental necroptosis in pregnant mice. A) Strategies for the in vivo experiments. B) Systolic blood pressure in the three groups (*n* = 6 per group) during pregnancy. C) The urine protein levels in each group were detected by ELISA kit. D) Representative photographs of fetuses from the three groups. E) Average fetal weight (*n* = 6 per group). F) Average placental weight (*n* = 6 per group). G) Representative Masson staining (scale bar = 500 µm), H&E staining (left panel: scale bar = 500 µm; right panel: scale bar = 50 µm), sFlt‐1 and VEGFA immunohistochemistry staining (scale bar = 50 µm) images of the mice placentae from each group. H) Representative IHC image of THBS1, TAK1 and NEDD4 in mice placentae from each group. Scale bar = 50 µm. I) Quantification of the IHC staining was performed using ImageJ software, *n* = 5 each group. ****, *p* < 0.0001. J,K). Western blot analysis of necroptosis and DAMPs protein levels in murine placentae from each group. *n* = 3 biologically independent experiments. *, L‐NAME versus PBS group. #, L‐NAME versus L‐NAME +THBS1 group. ***, ###, *p* < 0.001, ****, ####, *p* < 0.0001. NS, no significance.

In vivo models of PE were established by subcutaneously injecting pregnant mice with L‐NAME in this study. We found that the protein level of THBS1 in the placentae of the L‐NAME injection group was significantly observably down‐regulated (Figure S10, Supporting Information). To investigate the role of THBS1 in PE in vivo, an L‐NAME‐induced PE model was established. Pregnant mice were randomly divided into three groups: PBS, L‐NAME, and L‐NAME+THBS1. The specific experimental strategy is illustrated in **Figure** **7**A. THBS1 improved L‐NAME‐induced hypertension and urinary protein levels to some extent (Figure 7B,C). In our previous studies, we found that L‐NAME dramatically reduced fetal weight compared to the control group, which is also a characteristic of PE. Therefore, we examined the fetal weights of each group and found that the average fetal weights were significantly higher in the L‐NAME+THBS1 group than in the L‐NAME group (Figure 7D,E). However, there was no difference in the placental weight among the three groups (Figure 7F).

We also investigated the pathological changes in the placenta of each group because of the commonly admitted pertinence to PE and defective placental function. It has been reported that THBS1 could directly bind to transforming growth factor‐β1 (TGF‐β1),^[^ ^31^ ^]^ and it could regulate collagen formation and induce tissue fibrosis through interaction with TGF‐β1).^[^ ^32^ ^]^Therefore, Masson staining was used to detect whether THBS1 caused the side effect of placental fibrosis. But the results showed that there was no difference in collagen deposition in the placenta among the three groups. The mouse placenta comprises three distinct zones: the maternal decidua, the junctional zone (Jz), and the labyrinth (L) zone.^[^ ^33^ ^]^ The H&E staining results revealed that mice injected with L‐NAME had a large area of necrosis in the placental junction zone, which contained an abundance of trophoblasts. No necrosis was observed in the PBS group, and the necrotic area was diminished in the placenta of the L‐NAME+THBS1 group. Imbalanced placental angiogenesis, such as the elevated production of soluble fms‐like tyrosine kinase‐1 (sFlt‐1) and decreased levels of vascular endothelial growth factor A (VEGFA) in the placenta, also contribute to the development of PE.^[^ ^34^ ^]^ Compared with the PBS group, up‐regulation of sFlt‐1 and down‐regulation of VEGFA were observed in the placental labyrinth zone of the L‐NAME group, according to the IHC results. Moreover, supplementation with THBS1 reversed the imbalance in placental angiogenesis induced by L‐NAME (Figure 7G).

Then we further confirmed the expression of THBS1, TAK1 and NEDD4 of the three groups by IHC. We found that the expression of THBS1 and TAK1 were down‐regulated in the L‐NAME group, while the expression was reversed after supplementing THBS1. The expression of NEDD4 was opposite to that of THBS1 and TAK1 (Figure 7H,I). The features of necroptosis in the murine placentae of the three groups were validated by western blot. The results demonstrated that THBS1 supplementation decreased the expression of necroptosis‐ and DAMPs‐related proteins, which were activated by L‐NAME (Figure 7J,K). Altogether, these findings indicated that THBS1 ameliorated PE‐like symptoms and placental necroptosis in a mouse model of PE.

## 3 Discussion

THBS1 is a matricellular glycoprotein which forms homotrimers comprised of different domains.^[^ ^35^ ^]^ It is widely produced and secreted by various of cell types, including vascular smooth muscle cells, endothelial cells, fibroblasts and leukocytes.^[^ ^36^ ^]^ Pro‐survival activities of THBS1, such as promoting hemostasis and enhancing survival during infections, determine its essentiality in the human body.^[^ ^37^ ^]^ Loss of THBS1 in mice results in reduced survival, impaired host defense, and exaggerated neutrophil activation following acute intrapulmonary *Pseudomonas aeruginosa* infection.^[^ ^38^ ^]^ And it is an endogenous host‐protective molecule that suppresses inflammation by triggering the production of the anti‐inflammatory cytokine IL‐10 in macrophages during the resolution phase of experimental LPS‐induced lung injury.^[^ ^39^ ^]^ Notably, many studies have reported that maternal serum THBS1 is significantly decreased in patients with PE or HELLP syndrome and correlates with disease severity.^[^ ^9^, ^10^ ^]^ Despite this, the function of THBS1 in PE remains unexplored. In this study, we observed that THBS1 was mainly located in placental villous trophoblasts and identified THBS1 as a downregulated DEP of in severe PE placentae. Further exploration for mechanism via in vitro and in vivo experiments suggested that inhibition of THBS1 induced necroptosis in trophoblast cells.

In normal pregnancy, cytotrophoblasts migrate into the maternal uterine spiral arteries and penetrate deeply into the myometrium, eventually forming vascular sinuses at the maternal‐fetal interface to provide nutrients to the fetus. Impairment of trophoblast invasion of the uterine spiral arteries is a hallmark of PE and leads to narrow maternal vessels and relative placental ischemia.^[^ ^40^ ^]^ Trophoblast cell death primarily accounts for its impaired migratory and invasive abilities.^[^ ^41^ ^]^ Emerging evidence has also shown that trophoblasts in preeclamptic placentae undergo diverse programmed cell death processes, including apoptosis, pyroptosis, necroptosis, and ferroptosis.^[^ ^42^ ^]^ Necroptosis is a novel form of PCD that involves a series of morphological characteristics, including cell swelling, loss of membrane integrity, and leakage of DAMPs, that overlap with necrosis and apoptosis.^[^ ^14^ ^]^ However, the association between necroptosis and PE is still controversial. Hannan et al. identified the presence and localization of important mediators of the necroptotic pathway in the human placenta.^[^ ^43^ ^]^ Yu et al. found that the expression levels of RIPK1, RIPK3, and p‐MLKL were higher in preeclamptic placentae than in healthy placentae.^[^ ^44^ ^]^ Zhang et al. found an upregulation of PGAM5 and increased necroptosis‐relevant protein expression in placentae from preeclampsia pregnancies.^[^ ^45^ ^]^ But Cheng et al. found no evidence of necroptosis‐associated events in placental tissues from patients with early‐onset or late‐onset PE.^[^ ^46^ ^]^ We provide new evidence for the presence of necroptosis and found an increase in the expression of DAMPs molecules in both the human and mice preeclamptic placenta.

Importantly, we confirmed that THBS1 knockdown led to necroptosis and release of DAMPs from HTR8/SVneo cells. The RIPK1 inhibitor Nec‐1 and RIPK3 inhibitor GSK′872 could improve impaired cell viability and proliferation, ameliorate the increase in apoptosis and reduce the expression of necroptosis‐ and DAMPs‐related proteins induced by THBS1 knockdown in HTR8/Svneo cells. Interestingly, the apoptosis inhibitor Z‐VAD‐FMK was ineffective in rescuing the adverse conditions caused by sh‐THBS1. Apoptosis is considered as a non‐inflammatory type of cell death since it rarely induces unwanted tissue destruction and is usually not accompanied by DAMPs production.^[^ ^47^ ^]^ DAMPs and other cytokines can synergistically contribute to sterile inflammation in the placental microenvironment, triggering maternal systemic inflammation and endothelial dysfunction.^[^ ^18^ ^]^ Therefore, necroptosis is more closely related to PE pathogenesis because of the inflammatory characteristics of releasing DAMPs.

TAK1 plays an indispensable role in the regulation of cellular necroptosis.^[^ ^28^ ^]^ Malireddi et al. observed the formation of an RIPK1 kinase activity‐independent multiprotein cell death complex in TAK1‐deficient macrophages.^[^ ^21^ ^]^ Shim et al. deleted the TAK1 gene in mice to analyze its function in vivo and observed early embryonic lethality in these mice.^[^ ^48^ ^]^ Naito et al. also indicated the essential role of necroptosis in mediating neurovascular damage and hypoperfusion‐induced TAK1 deficiency.^[^ ^49^ ^]^ We also observed that the expression of TAK1 in preeclamptic placentae was downregulated compared to that in the placentae of normal pregnancies in this study. Here, we report for the first time that TAK1 could interact with THBS1, and its protein level was positively correlated with that of THBS1 in both HTR8/Svneo cells and human placental samples, suggesting that TAK1 may be a key upstream molecule in the necroptosis of trophoblast cells with low THBS1 expression. Notably, the N‐terminal domain of THBS1, which has been reported to bind to aggrecan, heparin, integrin, low‐density lipoprotein receptor‐related protein, and tumor necrosis factor‐stimulated gene‐6,^[^ ^50^ ^]^ had the most binding sites for TAK1. This suggests that the N‐terminal domain of THBS1 may be a potent therapeutic target for PE.

NEDD4 was proposed to act as an E3 ligase of TAK1 and catalyze the K48‐linked ubiquitination of TAK1 in HTR8/SVneo cells with THBS1 knockdown. Ubiquitination is an essential post‐translational modification that covalently links an evolutionarily conserved 76‐amino acid polypeptide, called ubiquitin, to target proteins.^[^ ^51^ ^]^ It has been shown to control basic biological functions, including cell survival, cell cycle, signal transduction, and transcriptional regulation.^[^ ^52^ ^]^ It is a highly orchestrated enzymatic cascade involved in multistep processes mediated by three classes of enzymes: ubiquitin‐activating enzymes (E1), ubiquitin‐conjugating enzymes (E2), and ubiquitin ligases (E3).^[^ ^53^ ^]^ Among them, E3 ubiquitin ligases are the most heterogeneous class of enzymes in the ubiquitination pathway and can determine substrate specificity by directly transferring the ubiquitin protein to the lysine site of targeted substrates.^[^ ^54^ ^]^ According to the characteristic domains and the mechanism of ubiquitin transfer to the substrate protein, E3 ligases can be classified into three types: RING E3s, HECT E3s, and RBR E3s.^[^ ^55^ ^]^ The HECT E3s in human contains 28 members, with the NEDD4 family being the largest group. NEDD4 family proteins have three functional domains: the N‐terminal C2 domain as a Ca^2+^ or phospholipid‐binding motif for membrane binding, the WW domain in the central region that interacts with PPXY motifs or phospho‐serine/threonine residues in substrates, and the HECT domain in the carboxyl terminus that promotes the transfer of ubiquitin to substrates.^[^ ^56^ ^]^ We found that NEDD4 could interact with TAK1, and the HECT domain of NEDD4 had the most binding sites for TAK1, which might be the main mechanism by which NEDD4 could reduce TAK1 protein levels by promoting K48‐linked polyubiquitination. K48‐linked polyubiquitination has been reported to negatively regulate the activation of TAK1; for example, FBXW2 degrades TAK1 via targeting TAK1 for K48‐linked polyubiquitination.^[^ ^57^ ^]^ In this study, NEDD4 knockdown in sh‐THBS1 cells reversed the expression of TAK1, necroptosis, and DAMPs molecules, confirming that activated NEDD4 is critical for regulating TAK1 and necroptosis in cells with THBS1 down‐regulation.

However, some limitations still existed in current investigation. First, since PE is a disease of placental origin, a conditional knockout mice of THBS1 in EVT may be a more suitable in vivo model. Secondly, this study found that THBS1 knockdown could induce pyroptosis of trophoblasts, suggesting that pyroptosis might be another non‐negligible mechanism of THBS1‐induced trophoblast dysfunction. Further experiments are required to determine the effect of THBS1 on trophoblast pyroptosis. Thirdly, although the overexpression of THBS1 could decrease the mRNA and protein levels of NEDD4 to stabilize the TAK1, it did not alter the cell cycle, apoptosis or the migratory and invasive abilities significantly in trophoblast cells. Therefore, THBS1 overexpression might have other effects on other signaling pathways which necessitates further exploration.

**Figure 8:**
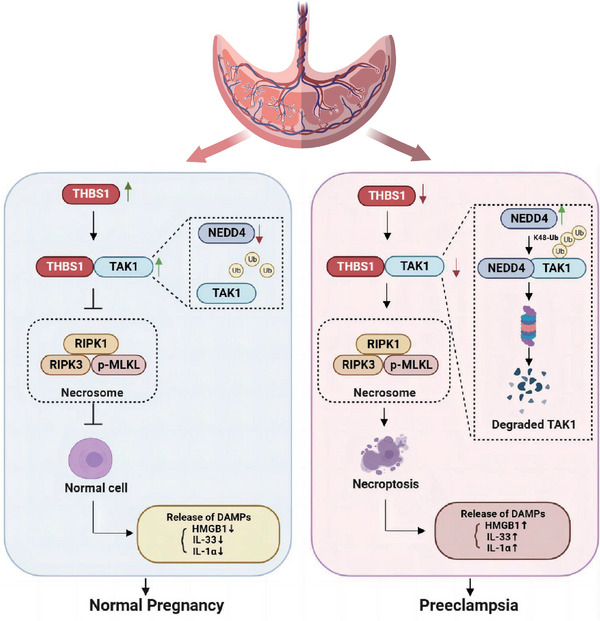
The down‐regulation of THBS1 could destabilize TAK1 via NEDD4‐mediated, K48‐linked TAK1 ubiquitination, resulting in the activation of the necroptosis and DAMPs release in trophoblast cells, thus participating in the pathogenesis of PE.

In summary, we identified that THBS1 is downregulated in severe preeclamptic placentae. Notably, we revealed a novel mechanism by which THBS1 knockdown induces necroptosis in trophoblasts through destabilizing TAK1 by the activation of NEDD4‐mediated, K48‐linked TAK1 polyubiquitination. In addition, the protective roles of THBS1 in a murine model of PE were uncovered. Altogether, these results expand our knowledge of the biological functions and clinical significance of THBS1 in PE and provide a therapeutic strategy for PE (**Figure** **8**).

## 4 Experimental Section

### Patients and Human Placenta Collection

This study was approved by the Ethics Committee of Nanfang Hospital, Southern Medical University, China (NFEC‐2020‐155). All enrolled patients provided signed informed consent. Patient information and placental samples were collected from the Department of Obstetrics and Gynecology at Nanfang Hospital, Southern Medical University. The inclusion criteria and procedures for placental collection were in accordance with those described in our previous study.^[^ ^58^ ^]^ In brief, the placentae were sliced with a sterile scalpel into three pieces of 1 cm^3^ cubes near the umbilical cord within 30 min after delivery. After washed with phosphate‐buffered saline (PBS) for several times, the collected tissues were stored in a −80 °C refrigerator or fixed in 4% paraformaldehyde for later experiments.

### Tandem Mass Tag (TMT)‐Based Quantitative Proteomics

Seven cryopreserved placental samples (three from patients with severe PE and four from patients with normal pregnancies) were sent to Novogene Co., Ltd. (Beijing, China) for TMT‐based quantitative proteomics. The total placental protein was extracted with a lysis buffer and quantified using a Bradford protein quantitative kit. Exactly 120 µg of each protein sample was digested and mixed with a TMT labeling reagent. After the separation of fractions, LC‐MS/MS analyses were performed using a Q Exactive HF‐X mass spectrometer (Thermo Fisher) equipped with an ion source of Nanospray Flex. The resulting spectra from each run were separately searched against the homo_sapiens_uniprot_2020_7_2. fasta (192 320 sequences) database using the search engine Proteome Discoverer 2.2 (PD 2.2, Thermo). After removing peptides and proteins with a false discovery rate higher than 1%, the protein quantitation results were statistically analyzed using a t‐test. Proteins with a *P*‐value <0.05 and fold change >1.5 or <0.67 were defined as differentially expressed proteins (DEPs). The DEPs were used for volcanic map analysis, cluster heat map analysis, and Gene Ontology (GO) and Kyoto Encyclopedia of Genes and Genomes (KEGG) enrichment analyses. All DEPs were uploaded as Table S1 (Supporting Information). Protein‐protein interaction (PPI) networks were predicted using STRING (http://string‐db.org/).

### Cell Culture and Treatment

The human trophoblast cell line HTR8/SVneo was purchased from the American Type Culture Collection (ATCC; Manassas, USA). HTR8/SVneo cells were cultured in RPMI‐1640 medium (Gibco, USA) supplemented with 10% fetal bovine serum (FBS; Gibco, USA) at 37 °C in a humidified incubator with 5% CO_2_ and 20% O_2_.

The lentiviral vector (Ubi‐MCS‐3FLAG‐SV40‐Neomycin) for human THBS1 overexpression was purchased from GeneChem (Shanghai, China). Moreover, the THBS1‐silencing lentiviral vectors (pds328_pL‐U6‐shRNA‐GFP‐ccdB‐Neomycin) were synthesized by Tsingke (Beijing, China). The short hairpin‐interfering RNA (shRNA) sequence was GTAGGTTATGATGAGTTTAAT. HTR8/SVneo cells were transfected with these lentiviruses according to the manufacturer's instructions. Then, the infected cells were selected in a complete medium containing 400 µg mL^−1^ G418 (Gibco, USA), and the surviving cells were used for subsequent experiments. The cells that expressed stable knockdown of THBS1 were treated with the RIPK1 inhibitor necrostatin‐1 (Nec‐1, 25 µm, MedChemExpress, USA), RIPK3 inhibitor GSK′872 (10 µm, MedChemExpress, USA), and pan‐caspase inhibitor Z‐VAD‐FMK (25 µm, MedChemExpress, USA) for 24 h.

The small interfering RNAs (siRNAs) of TAK1, NEDD4 and plasmids, including Flag‐tagged TAK1, Myc‐tagged NEDD4, and HA‐tagged wild‐type ubiquitin (HA‐Ub) and its mutants (HA‐Ub‐K48R and HA‐Ub‐K63R), were purchased from IGEbio (Guangzhou, China). The siRNA sequences of TAK1 and NEDD4 are presented in Table S2 (Supporting Information). Transfection of siRNAs and plasmids was performed using Lipofectamine 3000 (Invitrogen, USA) according to the manufacturer's instructions.

### Cell Counting Kit‐8 (CCK‐8) Assay

The viability of HTR8/SVneo cells was determined using a CCK‐8 kit (Dojindo, Japan). An appropriate number of cells (5 × 10^3^ or 1 × 10^4^) suspended in a 100‐µL complete medium were seeded into each well of a 96‐well plate and mixed with 10 µL of CCK‐8 reagent. After incubation at 37 °C for 2 h, the viability was measured at 450 nm using a microplate reader (BioTek, USA).

### 5‐Ethynyl‐2′‐Deoxyuridine (EdU) Assay

Cell proliferation was assessed using an EdU detection kit (RiboBio, Guangzhou, China) according to the manufacturer's protocol. Generally, after undergoing different treatments, the cells were exposed to a fresh medium containing 50 µM of EdU and cultured for another 2 h. The cells were fixed in 4% paraformaldehyde and permeabilized with 0.5% Triton X‐100. Then, they were incubated with 100 µl of Apollo 567 for 30 min, followed by Hoechst 33 342 staining for 5 min. After staining, the cells were observed under a fluorescence microscope (Leica, Germany). The cell proliferation rate was calculated as the ratio of the number of EdU‐incorporated cells (magenta) to the number of Hoechst 33342‐staining cells (blue).

### Cell Cycle and Apoptosis

Cell cycle analysis was performed using the Cell Cycle Detection Kit (KeyGEN, China). HTR8/Svneo cells were digested using 0.25% trypsin and resuspended in phosphate‐buffered saline (PBS). Then, the cell suspension was fixed in 75% ethanol at 4 °C overnight. The fixed cells were washed with PBS and incubated with RNase A and propidium iodide (PI). Subsequently, the cell cycle was assessed within 1 h using a flow cytometer (BD LSRFortessa, USA).

Cell apoptosis was detected using an Annexin V‐Light 650/PI Apoptosis Detection Kit (Wanleibio, China), according to the manufacturer's instructions. The supernatant and cells were harvested, washed, and suspended in 500 µL of a binding buffer. Then, 5 µL of Annexin V‐Light 650 was added into the cell suspension and mixed with PI. After 5–15 min of incubation in the dark, cell apoptosis was detected within 1 h using flow cytometry.

### Cell Migration and Invasion Assay

Cell culture inserts (8 µm pore size) of the transwell assay were purchased from Corning (USA) and placed in 24‐well plates. For the cell migration assay, the 200 µL cell suspensions with a serum‐free medium of HTR8/SVneo cells were seeded in the upper chambers. Then, 600 µL of the culture medium containing 10% FBS was added to the lower chambers as a chemo‐attractant.

For the cell invasion assay, the upper chambers were pre‐coated with 50 µl of Matrigel (BD, USA) before cell seeding. Matrigel was diluted in a serum‐free medium at a ratio of 1:8. After 24–48 h, the migratory and invasive cells were fixed with methanol for 30 min and stained with crystal violet (Beyotime, China) for 20 min. Images of stained cells were captured under a microscope.

### Animal Experiments

The animal experiments were approved by the Animal Ethical and Welfare Committee of the Guangdong Medical Laboratory Animal Center (approval number: C202210‐1). All the procedures were conducted in accordance with the National Institutes of Health Guide for the Care and Use of Laboratory Animals. C57BL/6J mice (8 weeks old) were purchased from the Guangdong Medical Laboratory Animal Center and housed under specific pathogen‐free (SPF) conditions with a 12‐h light‐dark cycle and ad libitum access to food and water. Female mice were mated with weight‐matched male mice overnight at a 2:1 ratio. The day on which pregnancy was confirmed by observing a vaginal plug was designated as the exact gestational day (GD 0.5).

As described in our previous study, subcutaneous injection of NG‐nitroarginine methyl ester hydrochloride (L‐NAME) was used to induce PE in the pregnant mice.^[^ ^59^, ^60^ ^]^ The pregnant mice were randomly assigned to three groups: PBS (*n* = 6), L‐NAME (*n* = 6), and L‐NAME + recombinant human THBS1 protein (*n* = 6). From GD 9.5 to GD 18.5, the L‐NAME and L‐NAME+THBS1 groups were subcutaneously injected with L‐NAME (125 mg kg^−1^ day^−1^, MedChemExpress, USA) and L‐NAME+THBS1 (0.5 mg kg^−1^ day^−1^, Novoprotein, China), respectively. The PBS group was simultaneously subcutaneously injected with an equal amount of PBS as the other two groups.

The systolic blood pressure (SBP) of pregnant mice was measured using a non‐invasive blood pressure monitor with a volume pressure‐recording sensor and an occlusion tail cuff (Softron Biotechnology, China). SBP measurements were performed on the day before administration and on GD 10.5, GD 14.5, and GD 18.5. Urine samples were collected on GD 18.5. Mouse urine protein ELISA kits (MEIMIAN, China) were used to detect proteinuria according to the manufacturer's instructions.

On GD 18.5, pregnant mice were anesthetized using pentobarbital, and their placentas and fetuses were removed. After obtaining photographs of the mouse fetus, the fetus and placentae were weighed separately. The murine placentae were either stored in a −80 °C refrigerator or quickly placed in 4% paraformaldehyde for subsequent experiments.

### RNA Extraction and Quantitative Real‐Time Polymerase Chain Reaction (RT‐qPCR)

Total RNA was extracted from treated HTR8/SVneo cells and human and mouse placental tissues using TRIzol Reagent (Life Technologies, USA). A NanoDrop spectrophotometer (Thermo Fisher Scientific, USA) was used to measure the RNA concentration and quality. Then, 1 µg of RNA from each sample was reverse transcribed into cDNA using a HiScript Q RT SuperMix for qPCR kit (Vazyme, China). RT‐qPCR was performed on a LightCycler 96 system (Roche, Switzerland) using ChamQ SYBR qPCR Master Mix (Vazyme, China). Relative mRNA levels were calculated using the 2–ΔΔCt method and normalized to the glyceraldehyde‐3‐phosphate dehydrogenase (GAPDH) level. The primers used are listed in Table S3 (Supporting Information).

### RNA Sequencing and Bioinformatics Analysis

RNA from HTR8/Svneo cells transfected with THBS1‐knockdown lentivirus (*n* = 3) and the corresponding empty vector (*n* = 3) was sent to Novogene Co., Ltd. (Beijing, China) for cDNA library construction, and being sequenced by the Illumina NovaSeq 6000. Raw RNA‐seq sequenced reads were aligned to the *Homo sapiens* reference genome using Hisat2 v2.0.5. software. FeatureCounts v1.5.0‐p3 was used to count the read numbers mapped to each gene. The expected number of Fragments Per Kilobase of transcript per Million mapped reads (FPKM) was calculated based on the length of the gene and the read count mapped to this gene. Differential gene expression was carried out with DESeq2 R package (1.20.0).and differentially expressed genes (DEGs) were defined as having an adjusted *p*‐value < 0.05, a log2 fold change>1. All DEGs were uploaded as Table S4 (Supporting Information). Enrichment analysis of DEGs was performed using Gene Ontology (GO) and the Kyoto Encyclopedia of Genes and Genomes (KEGG) analysis using the clusterProfiler R package. Gene set enrichment analysis (GSEA) was performed to analyze the ranked lists of all available genes.

### Western Blot

Total protein was extracted from treated HTR8/SVneo cells and human and mouse placental tissues using RIPA with phosphatase and protease inhibitors. Protein concentrations were quantified using the BCA Protein Assay Kit (Beyotime, China). The extracted proteins were separated using 8% or 10% sodium dodecyl sulfate‐polyacrylamide gel electrophoresis (SDS‐PAGE) and transferred onto polyvinylidene difluoride (PVDF) membranes. After blocking with 5% skim milk diluted in tris‐buffered saline (TBS), the membranes were incubated with the primary antibodies at 4 °C overnight. The antibodies used in this study are listed in Table S5 (Supporting Information). On the following day, the membranes were washed with TBST and incubated with the corresponding secondary antibodies for 2 h. Finally, the membranes were soaked in an ECL substrate working solution (Abbkine, China) and subsequently placed in an imaging system (Tanon, China) to detect the protein bands. The protein levels were then quantified via ImageJ software. GAPDH was used as the loading control.

### Co‐Immunoprecipitation

Co‐immunoprecipitation (Co‐IP) assays were performed using the Pierce Classic Magnetic IP/Co‐IP Kit (Thermo Scientific, USA). The transfected and treated HTR8/SVneo cells were lysed using IP lysis. The lysates were centrifuged at 13 000 g for 10 min to pellet the cell debris, and the supernatants of the samples were incubated with the indicated antibodies at 4 °C overnight. The following day, the antigen‐antibody complex was bound to the protein A/G magnetic beads for 1 h at room temperature, followed by washing the beads twice with IP wash buffer and once with purified water. After eluting the antigen‐antibody complex from the beads, the IP products were analyzed using western blot.

### Cycloheximide (CHX) Chase Assay

A cycloheximide (CHX) chase assay was conducted to monitor the half‐life of the proteins. In general, the cells were treated with the protein synthesis inhibitor CHX (100 µg mL^−1^) for 0 h, 3 h, 6 h, and 9 h. After harvesting the proteins at each time point, western blot was performed to detect the protein expression.

### Hematoxylin‐Eosin (H&E) Staining, Masson's Trichrome Staining, Immunohistochemistry (IHC), and Immunofluorescence

Human and mouse placentas were fixed overnight in 4% paraformaldehyde. Then, the tissues were dehydrated, embedded in paraffin, and cut into 4 µm‐thick serial sections for subsequent experiments. The sections were stained with H&E and viewed under a microscope. The nuclei were stained blue, and the cytoplasm was stained red.

For Masson's trichrome staining, sections of the mouse placentae were dewaxed and rehydrated using xylene and a gradient of ethanol. The nuclei were stained with Weigert hematoxylin, and the cytoplasm or red blood cells were stained with Ponceau magenta. Then, the sections were treated with 1% phosphomolybdic acid solution, and the collagen fibers were stained with aniline blue. After treatment with 1% glacial acetic acid and repeated dehydration with 95% alcohol, the sections were dehydrated until sufficiently transparent for examination.

For IHC, the sections were deparaffinized, rehydrated, and incubated with sodium citrate buffer (10 mM, pH 6.0) at a high temperature to retrieve the antigens. The sections were then incubated with 3% hydrogen peroxide for 20 min to inactivate endogenous peroxidase activity and blocked with 1% goat serum albumin at room temperature for 20 min. After incubation with the indicated primary antibody overnight at 4 °C, the sections were incubated with the corresponding secondary antibody at room temperature for 30 min. Then, the sections were washed with TBS, stained with the DAB working reagent, and counterstained with hematoxylin. Finally, images were obtained under a microscope. The average optical density of each image was measured using ImageJ software.

Immunofluorescence was performed using fixed HTR8/SVneo cells and human placental sections. This assay was performed as previously described.^[^ ^61^ ^]^

### AlphaFold2 Structure Prediction and Visualization

To predict the interaction between THBS1‐TAK1 and NEDD4‐TAK1, we established the following pipeline. Owing to the massive Video Random Access Memory needed for AlphaFold2 predictions, the THBS1 sequence was divided into six segments and the NEDD4 sequence into three segments based on their different domains. These segments were matched with full‐length TAK1 to form individual groups for AlphaFold2 complex structure prediction. For each AlphaFold2 prediction, the full BFD database was used for sequence alignment, and each prediction took several hours to run on NVIDIA RTX 3080. Five structures were predicted for each group and ranked the resulting structures using pLDDTs. The top‐ranked complex structure was selected for further analysis. In the further analysis, the Measurement plugin was used in PyMOL 2.5.4 to analyze the complex interfaces. The analyzed interfaced graph was output using the draw command in PyMOL and further labeled using Abobe Illustrator 2017.

### Statistical Analysis

SPSS v20.0 software (IBM, USA) and GraphPad Prism v9.0 software (GraphPad, USA) were used for statistical analysis. All data are presented as mean ± standard deviation (SD) of at least three independent experiments. The normality of the data was assessed using the Shapiro‐Wilk test. Student's *t*‐test (for data conforming to a normal distribution) or Mann‐Whitney U test (for data not conforming to a normal distribution) was used to compare two groups of independent samples. Multiple comparisons were performed using a one‐way analysis of variance, followed by a homogeneity of variance test. If the results conformed to the homogeneity of variance, pairwise comparisons between groups were assessed using the least significant difference method; otherwise, they were assessed using Dunnett's *t*‐test. The Pearson correlation test (for data conforming to a normal distribution) or the Spearman correlation test (for data not conforming to a normal distribution) was used for correlation analysis. The level of statistical significance was set at *p* < 0.05.

## Conflict of Interest

The authors declare no conflict of interest.

## Supporting information

Supporting Information

Supplemental Table 1

Supplemental Table 4

## Data Availability

The data that support the findings of this study are available from the corresponding author upon reasonable request.

## References

[1] B. Mol , C. T. Roberts , S. Thangaratinam , L. A. Magee , C. de Groot , G. J. Hofmeyr , Lancet 2016, 387, 999. 26342729 10.1016/S0140-6736(15)00070-7

[2] Obstet Gynecol 2020, 135, 1492. 32443077 10.1097/AOG.0000000000003892

[3] C. Apicella , C. Ruano , C. Mehats , F. Miralles , D. Vaiman , Int. J. Mol. Sci. 2019, 20, 2837. 31212604 10.3390/ijms20112837 PMC6600551

[4] G. J. Burton , C. W. Redman , J. M. Roberts , A. Moffett , BMJ 2019, 366, l2381. 31307997 10.1136/bmj.l2381

[5] N. L. Baenziger , G. N. Brodie , P. W. Majerus , Proc Natl Acad Sci USA 1971, 68, 240. 5276296 10.1073/pnas.68.1.240 PMC391203

[6] A. Aburima , M. Berger , B. Spurgeon , B. A. Webb , K. S. Wraith , M. Febbraio , A. W. Poole , K. M. Naseem , Blood 2021, 137, 678. 33538796 10.1182/blood.2020005382

[7] Curr. Drug Targets 2008, 9, 863. 18855620 10.2174/138945008785909365 PMC3010394

[8] S. Kaur , S. M. Bronson , D. Pal‐Nath , T. W. Miller , D. R. Soto‐Pantoja , D. D. Roberts , Int. J. Mol. Sci. 2021, 22, 4570. 33925464 10.3390/ijms22094570 PMC8123789

[9] I. Ulu , Y. Cekmez , K. S. Yildirim , N. Ozer , E. E. Yogurtcuoglu , P. Angin , G. Kiran , J Matern Fetal Neonatal Med 2019, 32, 2543. 29471751 10.1080/14767058.2018.1441279

[10] B. Stenczer , A. Molvarec , G. Szabo , A. Szarka , G. Fugedi , J. Szijarto , J. J. Rigo , Thromb. Res. 2012, 129, 470. 22035632 10.1016/j.thromres.2011.09.032

[11] H. S. Chaouhan , C. Vinod , N. Mahapatra , S. H. Yu , I. K. Wang , K. B. Chen , T. M. Yu , C. Y. Li , Int. J. Mol. Sci. 2022, 23, 12714. 36361505 10.3390/ijms232112714 PMC9655262

[12] A. Murao , M. Aziz , H. Wang , M. Brenner , P. Wang , Apoptosis 2021, 26, 152. 33713214 10.1007/s10495-021-01663-3 PMC8016797

[13] S. J. Martin , FEBS J. 2016, 283, 2599. 27273805 10.1111/febs.13775

[14] M. E. Choi , D. R. Price , S. W. Ryter , A. Choi , JCI Insight 2019, 4, e128834. 31391333 10.1172/jci.insight.128834 PMC6693822

[15] H. Yu , L. Chen , B. Du , Cell Cycle 2023, 22, 1713. 37365800 10.1080/15384101.2023.2229138 PMC10446795

[16] A. C. Harmon , D. C. Cornelius , L. M. Amaral , J. L. Faulkner , M. J. Cunningham , K. Wallace , B. Lamarca , Clin. Sci. 2016, 130, 409. 10.1042/CS20150702 PMC5484393 26846579

[17] S. Girard , A. E. Heazell , H. Derricott , S. M. Allan , C. P. Sibley , V. M. Abrahams , R. L. Jones , Am. J. Reprod. Immunol. 2014, 72, 422. 24867252 10.1111/aji.12274 PMC4369138

[18] S. Banerjee , Z. Huang , Z. Wang , A. Nakashima , S. Saito , S. Sharma , S. Cheng , Front Cell Infect Microbiol 2021, 11, 694298. 34485175 10.3389/fcimb.2021.694298 PMC8415471

[19] K. Yamaguchi , K. Shirakabe , H. Shibuya , K. Irie , I. Oishi , N. Ueno , T. Taniguchi , E. Nishida , K. Matsumoto , Science 1995, 270, 2008. 8533096 10.1126/science.270.5244.2008

[20] A. A. Ajibade , H. Y. Wang , R. F. Wang , Trends Immunol. 2013, 34, 307. 23664135 10.1016/j.it.2013.03.007

[21] R. Malireddi , P. Gurung , S. Kesavardhana , P. Samir , A. Burton , H. Mummareddy , P. Vogel , S. Pelletier , S. Burgula , T. D. Kanneganti , J. Exp. Med. 2020, 217, 20191644. 10.1084/jem.20191644 PMC7062518 31869420

[22] X. Guo , H. Yin , Y. Chen , L. Li , J. Li , Q. Liu , Cell Death Dis. 2016, 7, e2381. 27685625 10.1038/cddis.2016.294 PMC5059887

[23] J. Yang , P. Sun , X. Xu , X. Liu , L. Lan , M. Yi , C. Xiao , R. Ni , Y. Fan , Aging Dis 2023, 14, 1799. 37196118 10.14336/AD.2023.0219 PMC10529759

[24] Y. R. Xu , C. Q. Lei , Front Immunol 2020, 11, 608976. 33469458 10.3389/fimmu.2020.608976 PMC7813674

[25] N. Foot , T. Henshall , S. Kumar , Physiol. Rev. 2017, 97, 253. 27932395 10.1152/physrev.00012.2016

[26] Y. Liu , Y. Chen , C. Ding , X. Zhu , X. Song , Y. Ren , Q. Wang , Y. Zhang , X. Sun , Int. J. Biol. Macromol. 2022, 219, 571. 35952808 10.1016/j.ijbiomac.2022.08.019

[27] Y. Liu , Y. Sun , S. Han , Y. Guo , Q. Tian , Q. Ma , S. Zhang , Cell Death Discov 2021, 7, 246. 34535633 10.1038/s41420-021-00637-3 PMC8448743

[28] R. Malireddi , S. Kesavardhana , T. D. Kanneganti , Front Cell Infect Microbiol 2019, 9, 406. 31850239 10.3389/fcimb.2019.00406 PMC6902032

[29] H. Koehler , S. Cotsmire , T. Zhang , S. Balachandran , J. W. Upton , J. Langland , D. Kalman , B. L. Jacobs , E. S. Mocarski , Cell Host Microbe 2021, 29, 1266. 34192517 10.1016/j.chom.2021.05.009 PMC9333947

[30] Y. T. Kwon , A. Ciechanover , Trends Biochem. Sci. 2017, 42, 873. 28947091 10.1016/j.tibs.2017.09.002

[31] L. Yao , F. Lu , S. Koc , Z. Zheng , B. Wang , S. Zhang , T. Skutella , G. Lu , Adv. Sci. (Weinh) 2023, 10, e2303711. 37672887 10.1002/advs.202303711 PMC10602550

[32] S. Rosini , N. Pugh , A. M. Bonna , D. Hulmes , R. W. Farndale , J. C. Adams , Sci Signal 2018, 11. 10.1126/scisignal.aar2566 29844053

[33] J. Rossant , J. C. Cross , Nat. Rev. Genet. 2001, 2, 538. 11433360 10.1038/35080570

[34] W. Dymara‐Konopka , M. Laskowska , A. Blazewicz , Curr. Pharm. Biotechnol. 2018, 19, 797. 30255753 10.2174/1389201019666180925115559

[35] J. C. Adams , Int. J. Biochem. Cell Biol. 1997, 29, 861. 9304800 10.1016/s1357-2725(96)00171-9

[36] S. M. Krishna , J. Golledge , Int. J. Cardiol. 2013, 168, 692. 23664438 10.1016/j.ijcard.2013.04.139

[37] S. Kaur , D. D. Roberts , J Cell Commun Signal 2023, 17, 485. 36689135 10.1007/s12079-023-00722-5 PMC10409698

[38] Y. Qu , T. Olonisakin , W. Bain , J. Zupetic , R. Brown , M. Hulver , Z. Xiong , J. Tejero , R. M. Shanks , J. M. Bomberger , V. S. Cooper , M. E. Zegans , H. Ryu , J. Han , J. Pilewski , A. Ray , Z. Cheng , P. Ray , J. S. Lee , JCI Insight 2018, 3, e96914. 29415890 10.1172/jci.insight.96914 PMC5821195

[39] Y. Zhao , Z. Xiong , E. J. Lechner , P. A. Klenotic , B. J. Hamburg , M. Hulver , A. Khare , T. Oriss , N. Mangalmurti , Y. Chan , Y. Zhang , M. A. Ross , D. B. Stolz , M. R. Rosengart , J. Pilewski , P. Ray , A. Ray , R. L. Silverstein , J. S. Lee , Mucosal Immunol. 2014, 7, 440. 24045574 10.1038/mi.2013.63 PMC3945733

[40] S. Rana , E. Lemoine , J. P. Granger , S. A. Karumanchi , Circ. Res. 2019, 124, 1094. 30920918 10.1161/CIRCRESAHA.118.313276

[41] A. W. Lokeswara , R. Hiksas , R. Irwinda , N. Wibowo , Front Cell Dev Biol 2021, 9, 726513. 34805141 10.3389/fcell.2021.726513 PMC8602860

[42] Y. Shan , C. Guan , J. Wang , W. Qi , A. Chen , S. Liu , Biomed. Pharmacother. 2023, 167, 115466. 37729725 10.1016/j.biopha.2023.115466

[43] N. J. Hannan , S. Beard , N. K. Binder , K. Onda , T. J. Kaitu'U‐Lino , Q. Chen , L. Tuohey , M. De Silva , S. Tong , Placenta 2017, 51, 1. 28292463 10.1016/j.placenta.2017.01.002

[44] H. Yu , Y. Zhang , M. Liu , L. Liao , X. Wei , R. Zhou , Placenta 2022, 120, 1. 35150983 10.1016/j.placenta.2022.01.014

[45] J. Zhang , J. Huang , X. Lin , K. Fei , Y. Xie , Q. Peng , X. Li , L. Xie , L. Dai , W. Zhang , Am. J. Reprod. Immunol. 2022, 87, e13539. 35304783 10.1111/aji.13539

[46] S. B. Cheng , A. Nakashima , W. J. Huber , S. Davis , S. Banerjee , Z. Huang , S. Saito , Y. Sadovsky , S. Sharma , Cell Death Dis. 2019, 10, 927. 31804457 10.1038/s41419-019-2162-4 PMC6895177

[47] A. Mazlo , Y. Tang , V. Jenei , J. Brauman , H. Yousef , A. Bacsi , G. Koncz , Int. J. Mol. Sci. 2022, 16, 24. 10.3390/ijms24010016 PMC9819908 36613458

[48] J. H. Shim , C. Xiao , A. E. Paschal , S. T. Bailey , P. Rao , M. S. Hayden , K. Y. Lee , C. Bussey , M. Steckel , N. Tanaka , G. Yamada , S. Akira , K. Matsumoto , S. Ghosh , Genes Dev. 2005, 19, 2668. 16260493 10.1101/gad.1360605 PMC1283960

[49] M. G. Naito , D. Xu , P. Amin , J. Lee , H. Wang , W. Li , M. Kelliher , M. Pasparakis , J. Yuan , Proc Natl Acad Sci USA 2020, 117, 4959. 32071228 10.1073/pnas.1916427117 PMC7060720

[50] A. Resovi , D. Pinessi , G. Chiorino , G. Taraboletti , Matrix Biol. 2014, 37, 83. 24476925 10.1016/j.matbio.2014.01.012

[51] D. Komander , M. Rape , Annu. Rev. Biochem. 2012, 81, 203. 22524316 10.1146/annurev-biochem-060310-170328

[52] A. Hershko , A. Ciechanover , Annu. Rev. Biochem. 1998, 67, 425. 9759494 10.1146/annurev.biochem.67.1.425

[53] C. M. Pickart , Annu. Rev. Biochem. 2001, 70, 503. 11395416 10.1146/annurev.biochem.70.1.503

[54] Q. Yang , J. Zhao , D. Chen , Y. Wang , Mol Biomed 2021, 2, 23. 35006464 10.1186/s43556-021-00043-2 PMC8607428

[55] F. E. Morreale , H. Walden , Cell 2016, 165, 248. 27015313 10.1016/j.cell.2016.03.003

[56] X. Lu , H. Xu , J. Xu , S. Lu , S. You , X. Huang , N. Zhang , L. Zhang , Front Physiol 2022, 13, 968927. 36091384 10.3389/fphys.2022.968927 PMC9458852

[57] S. Xia , L. Ji , L. Tao , Y. Pan , Z. Lin , Z. Wan , H. Pan , J. Zhao , L. Cai , J. Xu , X. Cai , Cell Mol. Gastroenterol. Hepatol. 2021, 12, 1121. 33962073 10.1016/j.jcmgh.2021.04.016 PMC8350196

[58] H. Hu , J. Jiang , Q. Chen , S. Wei , M. Liu , X. Chen , C. Fan , J. Ma , W. Chen , X. Wang , M. Zhong , Life Sci. 2020, 261, 118351. 32858039 10.1016/j.lfs.2020.118351

[59] H. Hu , W. Chen , Z. Tao , Z. Li , J. He , Y. Peng , J. Ma , H. Wen , J. Li , X. Wang , M. Zhong , Placenta 2022, 117, 95. 34785431 10.1016/j.placenta.2021.11.003

[60] J. Ma , H. Hu , M. Lin , L. Chen , M. Liu , H. Li , S. Quan , Placenta 2021, 106, 30. 33610935 10.1016/j.placenta.2021.02.002

[61] H. Hu , J. Ma , Z. Li , Z. Ding , W. Chen , Y. Peng , Z. Tao , L. Chen , M. Luo , C. Wang , X. Wang , J. Li , M. Zhong , Mol. Cell. Endocrinol. 2022, 548, 111614. 35304192 10.1016/j.mce.2022.111614

