# Supplementary material for: Thrombospondin‐1 Regulates Trophoblast Necroptosis via NEDD4‐Mediated Ubiquitination of TAK1 in Preeclampsia
Source: Adv Sci (Weinh). 2024 Apr 3;11(21):2309002. doi: 10.1002/advs.202309002 (PMC11151050; doi:10.1002/advs.202309002)

ADVANCED SCIENCE
Open Access

Supporting Information

for *Adv. Sci.*, DOI 10.1002/advs.202309002

Thrombospondin-1 Regulates Trophoblast Necroptosis via NEDD4-Mediated Ubiquitination of TAK1 in Preeclampsia

Haoyue Hu, Jing Ma, You Peng, Rixuan Feng, Chenling Luo, Minyi Zhang, Zixin Tao, Lu Chen, Tao Zhang, Wenqian Chen, Qian Yin, Jinguo Zhai, Jun Chen, Ailan Yin, Chi Chiu Wang* and Mei Zhong**

Supplementary Materials

Table S2. The siRNA sequences of NEDD4 and TAK1.

Name	Forward (5’-3’)	Reverse (5’-3’)
si-NC	UUCUCCGAACGUGUCACGUdTdT	ACGUGACACGUUCGGAGAAdTdT
si-NEDD4-1	GGAUUGAGUUUGAUGGUGAdTdT	UCACCAUCAAACUCAAUCCdTdT
si-NEDD4-2	GCAAAUGGCUGCUUUUAAAdTdT	UUUAAAAGCAGCCAUUUGCdTdT
si-NEDD4-3	CCAUGAAUCUAGAAGAACAdTdT	UGUUCUUCUAGAUUCAUGGdTdT
si-TAK1-1	CCCGUGUGAACCAUCCUAAUAdTdT	UAUUAGGAUGGUUCACACGGGdTdT
si-TAK1-2	CAGUGUGUCUUGUGAUGGAAUdTdT	AUUCCAUCACAAGACACACUGdTdT
si-TAK1-3	GACACACAUGACCAAUAACAAdTdT	UUGUUAUUGGUCAUGUGUGUCdTdT

Table S3 The primers for quantitative real- time PCR in this study.

Primer sequences for quantitative RT-qPCR		
Gene (Homo sapiens)	Forward	Reverse
THBS1	5’-AGCATCCGCAAAGTGACTGA -3’	5’-GGCAGGACACCTTTTTGCAG-3’
TAK1	5’-CTTCGGCAGTTATCCCGTGT-3’	5’-TTGCGTGGGCAGCAGTATAA-3’
NEDD4	5’-CACAAGCCTCCACCAGTGAT-3’	5’-TCCTCTCAGATGGGCTGGAA-3’
GAPDH	5’-AGCCACATCGCTCAGACAC-3’	5’-GCCCAATACGACCAAATCC-3’

Table S5. The antibodies used in this study.

The antibodies used in this study			
**NAME**	**Application**	**Source**	**Catalog**
THBS1	WB, IHC, IF	Proteintech, China	18304-1-AP
THBS1	IP	Abcam, UK	ab267388
HLA-G	IF	Proteintech, China	66447-1-Ig
CD31	IF	Abcam, UK	ab76533
RIPK1	WB, IHC	Proteintech, China	17519-1-AP
p-RIPK3	WB	Abcam, UK	ab209384
p-RIPK3	WB	Affinity, USA	#AF7443
RIPK3	WB, IHC	Proteintech, China	17563-1-AP
p-MLKL	WB, IHC	Affinity, USA	#AF7420
HMGB1	WB	Proteintech, China	10829-1-AP
IL-1α	WB	Proteintech, China	16765-1-AP
IL-33	WB	Proteintech, China	12372-1-AP
TAK1	WB, IHC, IF, IP	Proteintech, China	12330-2-AP
NEDD4	WB, IHC, IF, IP	Proteintech, China	21698-1-AP
Flag-Tag	WB, IP	Cell Signaling Technology, USA	#14793
HA-Tag	WB, IP	Cell Signaling Technology, USA	#3724
Myc-Tag	WB	Cell Signaling Technology, USA	#2278
sFlt-1	IHC	Abcam, UK	ab32152
VEGFA	IHC	Proteintech, China	19003-1-AP
GAPDH	WB	Proteintech, China	10494-1-AP

Figure S1. The efficiency of knockdown (sh-) or overexpression (OE-) of THBS1 with lentiviruses in HTR8/SVneo cells.

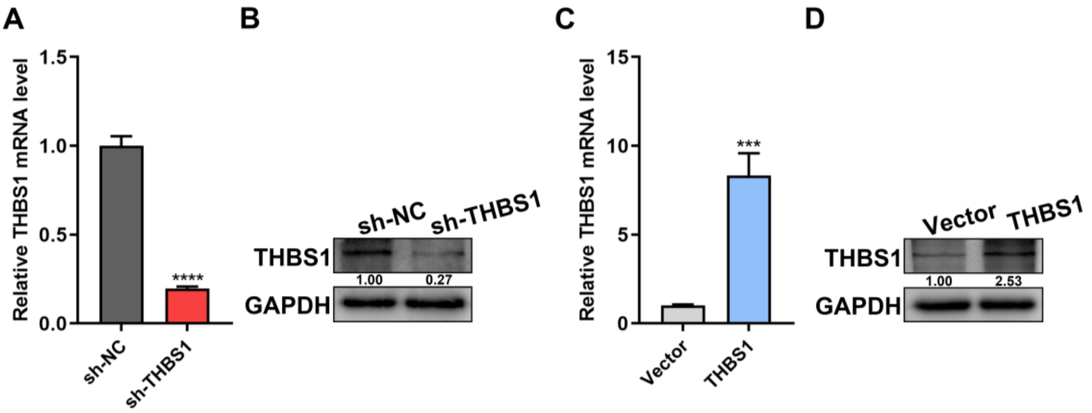

(A, C). RT-qPCR analysis of TAK1 mRNA expression in silencing or overexpressing THBS1 in HTR8/SVneo cells. n = 3 biologically independent experiments. (B-D). Western blot analysis of TAK1 protein level in silencing or overexpressing THBS1 in HTR8/SVneo cells. n = 3 biologically independent experiments. ***, $p < 0.001$, ****, $p < 0.0001$.

Figure S2. The Effects of overexpression of THBS1 on trophoblast cells.

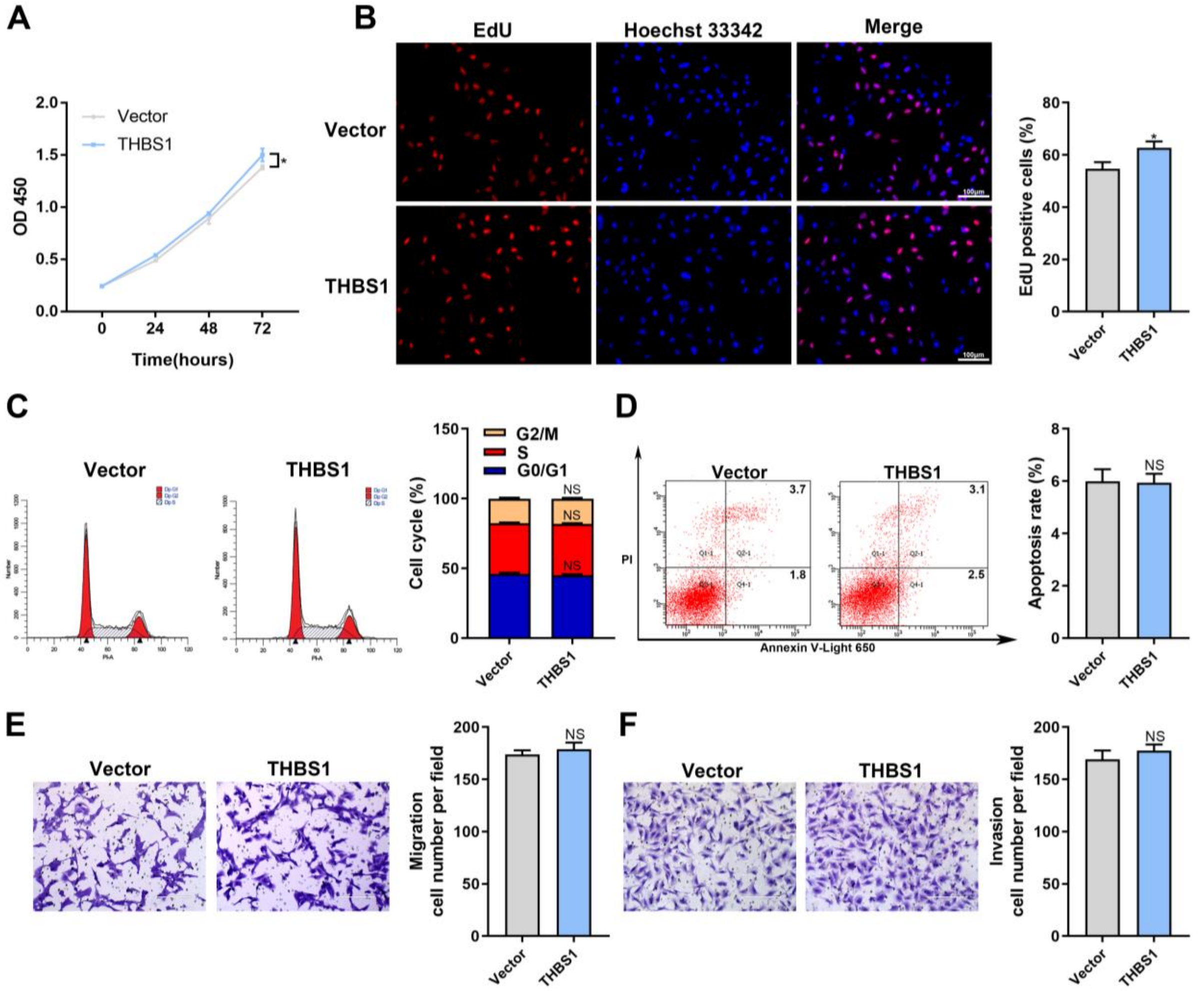

(A). CCK8 assay was used to detect the cell viability of Vector and OE-THBS1 stable-transfected

HTR8/SVneo cells at specific time points. n = 5 biologically independent experiments. (B). EdU assay was used to determine the proliferation of Vector and OE-THBS11 stable-transfected HTR8/SVneo cells. Left panel: representative images of the EdU assay in each group; right panel: statistics of EdU positive cells. n = 3 biologically independent experiments. (C). The cell cycle of Vector and OE-THBS1 stable-transfected HTR8/SVneo cells was measured by flow cytometry. Left panel: representative images of flow cytometry in each phase; right panel: quantification of cells in each phase. n = 3 biologically independent experiments. (D). Apoptosis of Vector and OE-THBS1 stable-transfected HTR8/SVneo cells was measured by Annexin V-Light 650/PI Apoptosis Detection Kit and flow cytometry. Left panel: representative histograms of apoptotic cells in each group; right panel: quantification of apoptotic cells in each group. n = 3 biologically independent experiments. (E). The migratory capability of Vector and OE-THBS1 stable-transfected HTR8/SVneo cells was examined by transwell assay. Left panel: representative images of migratory cells in each group; right panel: quantification of cells in each group. n = 3 biologically independent experiments. (F). The invasive capability of Vector and OE-THBS1 stable-transfected HTR8/SVneo cells was examined by transwell assay. Left panel: representative images of invasive cells in each group; right panel: quantification of cells in each group. n = 3 biologically independent experiments. *, $p < 0.05$, NS, no significance.

Figure S3. The protein levels of necroptosis and DAMPs in the clinical samples.

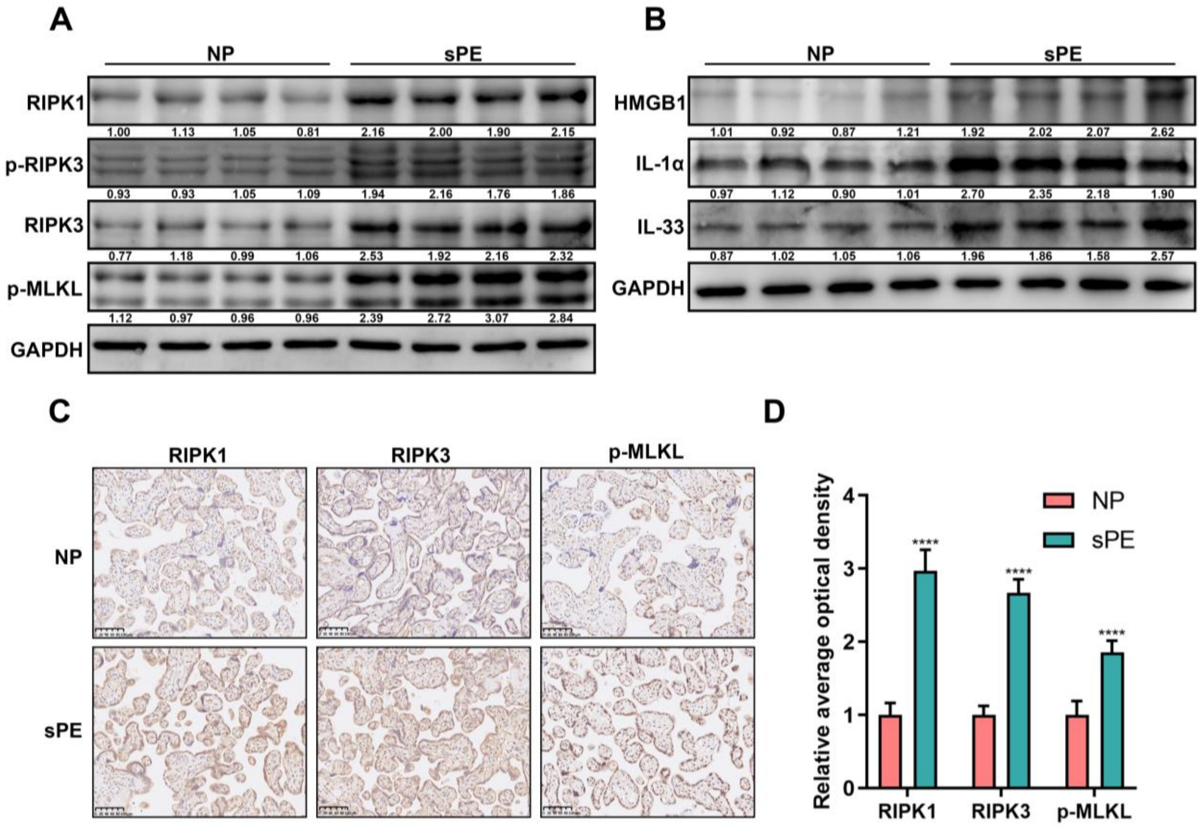

(A-B). Western blot analysis of necroptosis and DAMPs protein level in sPE and NP placentae. n = 3 biologically independent experiments. (C). Representative IHC image of RIPK1, RIPK3 and p-MLKL in sPE and NP placentae. Scale bar = 100 μm. (D). Quantification of the IHC staining was performed using

ImageJ software, n = 5 each group. ****, $p < 0.0001$.

Figure S4. The protein levels of pyroptosis (NLRP3, GSDMD, ASC and cleaved-caspase 1) in HTR8/SVneo cells that knockdown of THBS1.

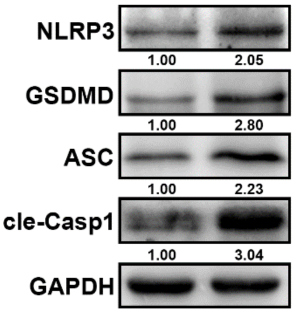

Figure S5. The expression of ZBP1 and TAK1 in the human placenta were examined via The Human Protein Atlas database.

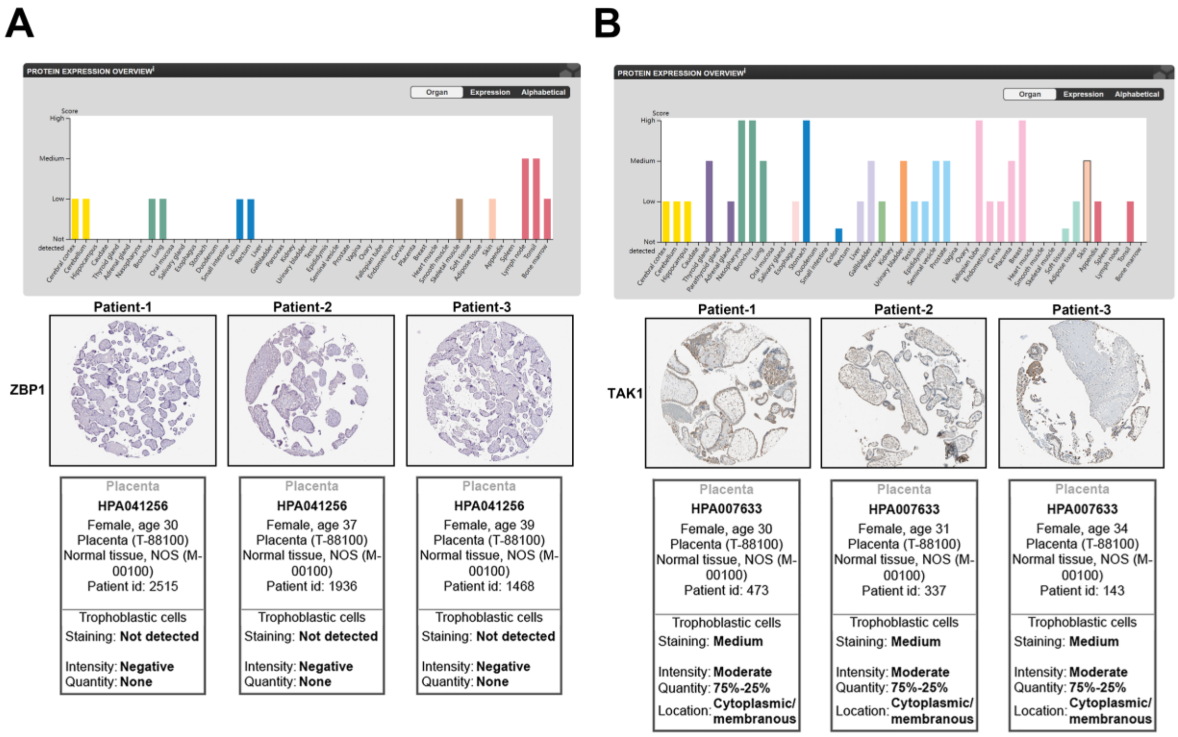

(A). The expression of ZBP1 in different human organs and placental IHC images of ZBP1. (B). The expression of TAK1 in different human organs and placental IHC images of ZBP1.

Figure S6. Inhibition of TAK1 induced necroptosis in trophoblast cells.

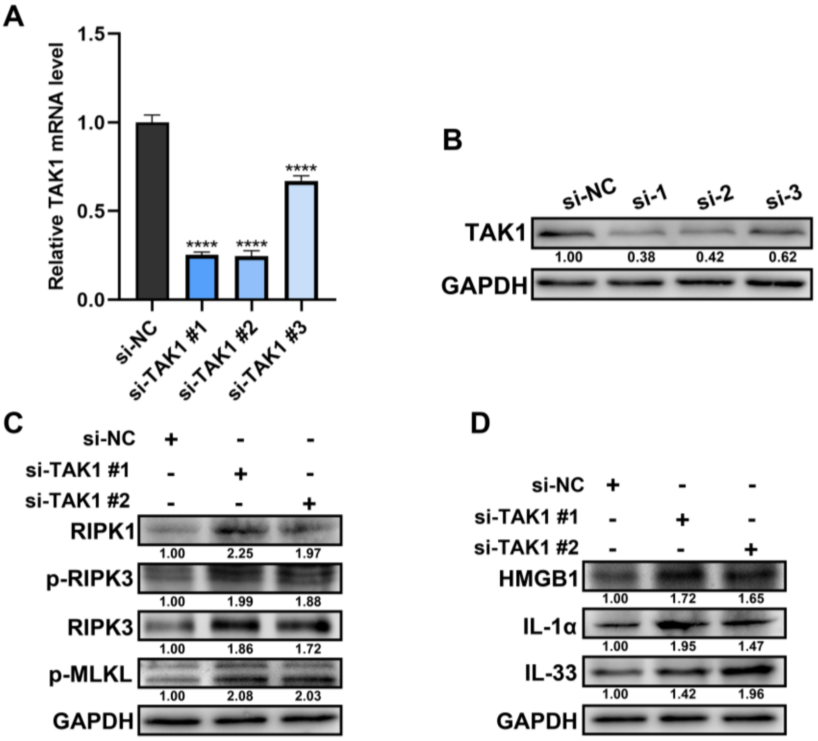

(A). RT-qPCR analysis of TAK1 mRNA expression in HTR8/SVneo cells that transfected with different siRNAs. n = 3 biologically independent experiments. (B). Western blot analysis of TAK1 protein level in HTR8/SVneo cells that transfected with different siRNAs. n = 3 biologically independent experiments. (C-D). After si-TAK1 transfection of HTR8/SVneo cells, western blot analysis of necroptosis and DAMPs protein levels was performed. n = 3 biologically independent experiments. ****, *p* < 0.0001.

Figure S7. The protein level of TAK1 in the clinical samples.

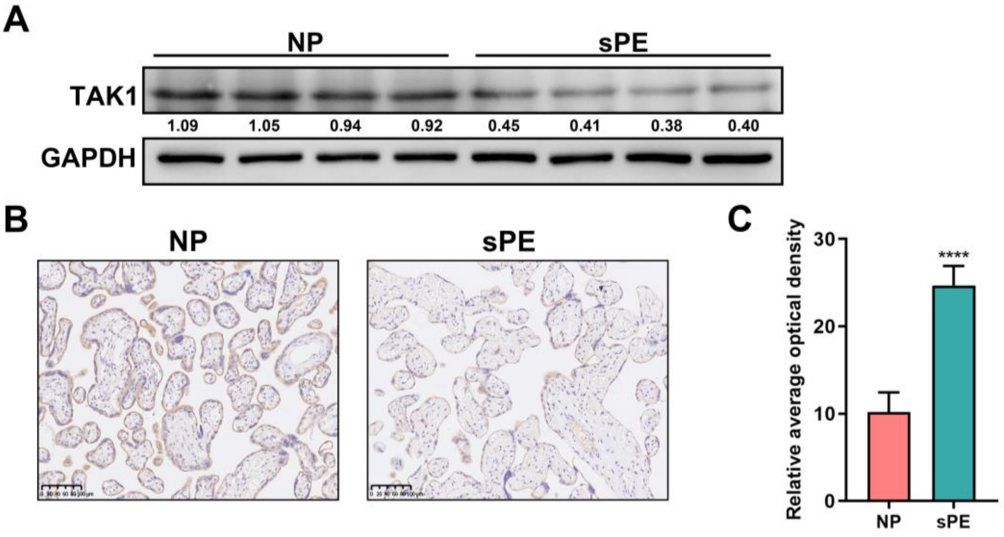

(A). Western blot analysis of TAK1 protein level in sPE and NP placentae. n = 3 biologically independent experiments. (B). Representative IHC image of TAK1 in sPE and NP placentae. Scale bar = 100 µm. (C). Quantification of the IHC staining was performed using ImageJ software, n = 5 each group. ****, *p* < 0.0001.

Figure S8. Top 10 E3 ubiquitin ligases that upregulated in the sh-THBS1 group in RNA-seq result.

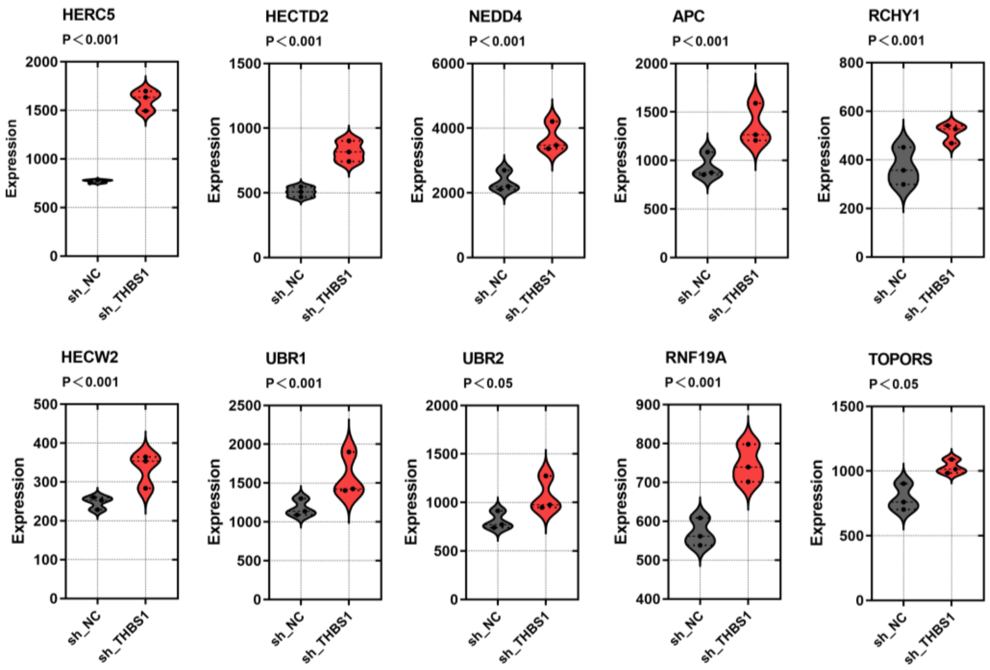

Figure S9. The knockdown efficiency of small interfering RNA (siRNA) of NEDD4.

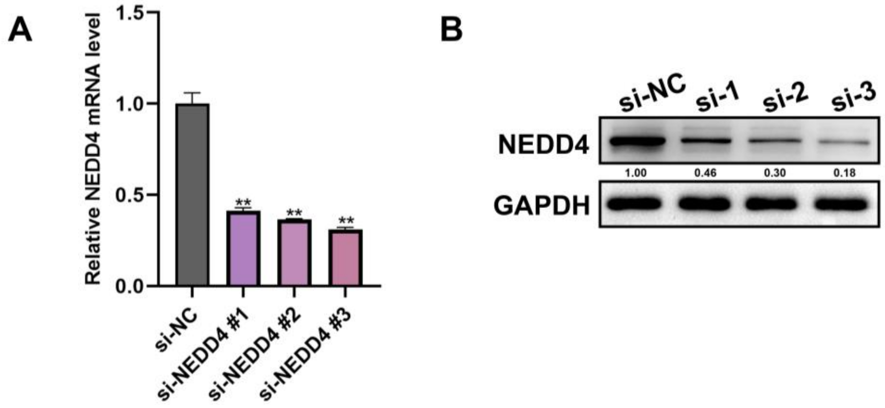

(A). RT-qPCR analysis of NEDD4 mRNA expression in HTR8/SVneo cells that transfected with different siRNAs. n = 3 biologically independent experiments. (B). Western blot analysis of NEDD4 protein level in HTR8/SVneo cells that transfected with different siRNAs. n = 3 biologically independent experiments. **, $p < 0.01$.

Figure S10. The protein level of THBS1 in placentae of PBS or L-NAME injection group.

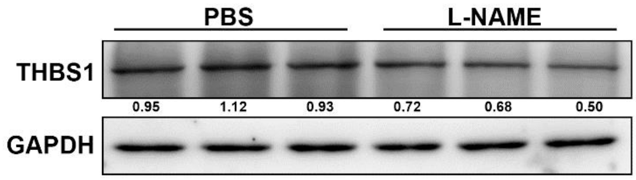

Supplement: Supplementary file 1 — Supporting Information [file ADVS-11-2309002-s002.pdf]
